# Biogeochemical Evaluation of Shilajit: Testosterone-Modulating Effects, In Vivo Toxicity and Lipid Profile Analysis

**DOI:** 10.3390/ph19091486

**Published:** 2026-09-17

**Authors:** Nodari Rizun, Vesna Stanković, Jovana Bradić, Marijana Anđić, Jelena Lađarević, Vladimir Jakovljević, Svitlana Zahorodnia, Nenad Janković

**Affiliations:** 1 Pürblack Inc., 41877 Enterprise Circle N., Temecula, CA 92590, USA; nodari@purblack.com; 2 Department of Pathology, Faculty of Medical Sciences, University of Kragujevac, 34000 Kragujevac, Serbia; vesna.stankovic@fmn.kg.ac.rs; 3 Department of Pharmacy, Faculty of Medical Sciences, University of Kragujevac, 34000 Kragujevac, Serbia; jovana.bradic@fmn.kg.ac.rs (J.B.); marijana.andjic@fmn.kg.ac.rs (M.A.); 4 Center of Excellence for Redox Balance Research in Cardiovascular and Metabolic Disorders, 69 Svetozara Markovića St., 34000 Kragujevac, Serbia; vladimir.jakovljevic@fmn.kg.ac.rs; 5 Faculty of Technology and Metallurgy, University of Belgrade, Karnegijeva 4, 11000 Belgrade, Serbia; jmirkovic@tmf.bg.ac.rs; 6 Department of Physiology, Faculty of Medical Sciences, University of Kragujevac, 69 Svetozara Markovića St., 34000 Kragujevac, Serbia; 7 Zabolotny Institute of Microbiology and Virology, The National Academy of Sciences of Ukraine, St. Academician Zabolotny, 154, 03143 Kyiv, Ukraine; svetazagorodnya@ukr.net; 8 Department of Science, Institute for Information Technologies Kragujevac, University of Kragujevac, Liceja Kneževine Srbije 1A, 34000 Kragujevac, Serbia

**Keywords:** urolithin A, antioxidant, testosterone, shilajit, mineral pitch, in vivo, HDL/LDL

## Abstract

**Background:** Shilajit is a complex mineral-organic resin derived from rocks of varied provenance, and it has been assessed here for its antioxidant activity and acute in vivo toxicity. **Methods:** Despite the increasing interest in shilajit as a dietary supplement, systematic data about its acute toxicity, biochemical safety, and biological effects remain limited. **Results:** Samples demonstrated moderate free radical scavenging ability, with IC_50_ values between 1.657 and 3.502 mg/mL for ABTS and 2.042 to 6.011 mg/mL for DPPH. Phenolic profiling identified samples **6** and **7** as the richest in urolithin A and phenolic acids. No fatalities, clinical signs of toxicity, or notable changes in food and water consumption or body weight gain were observed during the 14-day period, suggesting minimal single acute oral toxicity (LD_50_ > 2000 mg kg^−1^). Serum analysis revealed changes in lipid and testosterone profiles. In an exploratory follow-up of the two samples with the highest free testosterone values (**6** and **7**), total and free testosterone were increased approximately 2.5-fold and 3.5-fold, respectively, relative to the control group. **Conclusions:** The tested shilajit samples were tolerated under the conditions of this study, with no mortality or overt clinical toxicity during the 14-day observation period. Histopathological examination revealed mild to moderate degenerative and necrotic changes in hepatic and renal tissues, without hepatic fibrosis or pronounced renal interstitial lesions. These findings support satisfactory single acute oral tolerability under the study conditions but do not exclude tissue effects or establish their reversibility. Further studies are warranted to assess the long-term safety of standardized shilajit resin.

## 1. Introduction

Natural products have played a central role in the development of traditional and modern medicine, serving as a rich source of bioactive compounds with diverse pharmacological properties. In recent decades, there has been renewed scientific interest in natural substances derived from plants, minerals, and complex organic–inorganic matrices, driven by the growing demand for alternative and complementary therapies, as well as by increasing concerns regarding the long-term safety and side effects of synthetic drugs. Among these natural substances, mineral pitch occupies a unique position due to its complex chemical composition, long history of use in traditional medical systems, and broad spectrum of reported biological activities, particularly its antioxidant potential [1,2,3]. Mineral pitch (mummio, shilajit, Himalayan tears, asphaltum, etc.) is a naturally occurring, tar-like substance that exudes from rocks in high-altitude mountain ranges, primarily in the Himalayas, Altai, Caucasus, Andes, and other mountainous regions. It is formed over decades and centuries through the gradual decomposition of plant matter under specific geological, atmospheric and microbial conditions. Traditionally, mineral pitch has been used in Ayurvedic, Unani, and Tibetan medicine as a rejuvenating agent, adaptogen, and general health tonic. Classical Ayurvedic texts describe mineral pitch as a “rasayana,” a category of substances believed to promote longevity, vitality, and resistance to disease [4]. In contemporary contexts, mineral pitch is increasingly marketed as a dietary supplement and nutraceutical, often promoted for its purported benefits on energy metabolism, cognitive function, fertility, immune modulation, and aging-related disorders [5,6,7,8].

Despite its long-standing traditional use and growing popularity, systematic scientific evaluation of shilajit’s safety, toxicity, and biochemical effects remains limited and fragmented. This gap is particularly relevant given the increasing global consumption of mineral pitch-based products, which vary widely in source, processing methods, chemical composition, and purity [9]. As with many natural products, the assumption that traditional use equates to safety is not necessarily justified, especially when such substances are consumed chronically, at higher doses, or in vulnerable populations. Therefore, comprehensive toxicological and biochemical studies are essential to establish evidence-based safety profiles and to elucidate the mechanisms underlying shilajit’s biological activity [10].

Oxidative stress, defined as an imbalance between the production of reactive oxygen species (ROS) and the capacity of biological systems to neutralize them, is implicated in the pathogenesis of numerous diseases, including cardiovascular disorders, neurodegenerative diseases, metabolic syndrome, cancer, and aging-related dysfunctions [11,12]. Endogenous antioxidant defense systems—comprising enzymatic antioxidants such as superoxide dismutase, catalase, and glutathione peroxidase, as well as non-enzymatic antioxidants—play a crucial role in maintaining redox homeostasis. However, under pathological conditions or environmental stress, these systems may become overwhelmed, leading to oxidative damage to lipids, proteins, and nucleic acids. Natural antioxidants have attracted significant attention as potential therapeutic and preventive agents against oxidative stress–related disorders. Mineral pitch is particularly interesting in this context due to its high content of humic substances, including fulvic acid and humic acid, as well as a wide range of low-molecular-weight organic compounds, trace elements, and minerals. Fulvic acid, considered one of the principal bioactive components of mineral pitch, has been shown to possess antioxidant, metal-chelating, and free radical–scavenging properties. Additionally, mineral pitch contains phenolic compounds, dibenzo-α-pyrones, amino acids, and other redox-active molecules that may contribute synergistically to its antioxidant effects. One of the most frequently reported and scientifically investigated properties of mineral pitch is its antioxidant activity [13,14,15,16].

However, the same complexity that underlies shilajit’s biological activity also raises concerns regarding toxicity and biochemical safety. Mineral pitch is a naturally occurring mineral–organic matrix that accumulates heavy metals, environmental contaminants, and potentially toxic substances depending on its geographical origin and processing [17]. Reports of contamination with lead, mercury, arsenic, and other heavy metals have been documented in poorly purified or counterfeit mineral pitch products [17,18,19]. Furthermore, the presence of bioactive compounds capable of interacting with metabolic and endocrine pathways necessitates careful evaluation of potential adverse effects on vital organs and biochemical parameters.

In vivo animal models, particularly rodents, provide a valuable platform for investigating both the toxicological profile and biochemical impact of natural products such as mineral pitch. Serum biochemical analysis is a cornerstone of toxicological assessment, offering insight into liver and kidney function, lipid metabolism, oxidative status, and endocrine balance [20,21,22]. Parameters such as total cholesterol, triglycerides, HDL and LDL cholesterol, atherogenic indices, enzymatic markers, and hormone levels are commonly used to detect subtle metabolic disturbances that may not be evident through gross observation or histopathology alone. When combined with antioxidant assays, these analyses allow for a more comprehensive understanding of both safety and efficacy.

Previous experimental studies have reported that mineral pitch may exert protective effects against oxidative stress by enhancing endogenous antioxidant defenses and reducing lipid peroxidation [20,23]. Some investigations have indicated improvements in mitochondrial function, ATP production, and redox balance following mineral pitch administration [21]. Additionally, mineral pitch has been suggested to influence lipid metabolism, glucose homeostasis, and hormonal regulation, which may be relevant to its traditional use as a tonic and fertility-enhancing agent [24,25]. The moderate antioxidant, anticancer, or hyperalgesia-suppressing activity demonstrated by mineral pitch positions it as a promising candidate for further investigation in anticancer therapy [26,27]. Nevertheless, these findings are often based on limited sample sizes, heterogeneous experimental designs, or in vitro models that do not fully reflect systemic effects.

The lack of standardized mineral pitch preparations complicates comparisons across studies and highlights the need for well-characterized samples in experimental research. Another critical aspect of mineral pitch research is its interaction with lipid metabolism and cardiovascular risk factors. Application of mineral pitch can modulate lipid profiles or reduce oxidative damage to LDL particles in non-alcoholic fatty liver disease, implying its hepatoprotective potential [28]. In addition to lipid metabolism, endocrine effects—particularly those related to androgen levels—have been reported in association with mineral pitch use. Traditional medicine attributes aphrodisiac and fertility-enhancing properties to mineral pitch, and some experimental and clinical studies have observed changes in testosterone levels following supplementation [29,30,31]. While such effects may be beneficial in certain contexts, they also raise questions regarding hormonal balance, feedback mechanisms, and long-term safety. Serum hormone measurements, therefore, represent an important component of biochemical toxicity assessment.

Overall, these findings are consistent with acceptable subchronic safety within certain dose ranges, but they also point to potential organ-specific effects that warrant further targeted investigation [32,33,34,35]. The present study is designed to address these issues by conducting an acute toxicity study in vivo, histopathology, and biochemical analysis, with particular emphasis on its antioxidant activity. Using an in vivo model, the effects of mineral pitch administration were evaluated on a panel of serum biochemical parameters relevant to lipid metabolism, cardiovascular risk, and endocrine balance. By integrating toxicological assessment with biochemical and antioxidant analyses, this work seeks to provide a more comprehensive understanding of mineral pitch’s biological profile. Such an approach is essential not only for validating traditional claims but also for identifying potential risks associated with its use.

## 2. Results

### 2.1. Antioxidant Activity Evaluation

The results of the antioxidant activity of the samples, evaluated by both assays, are summarized in Table 1 as IC_50_ values and TEAC. The IC_50_ value represents the concentration required to achieve 50% of the maximum activity, with lower values indicating stronger antioxidant potential. Comparison with the reference standards revealed that the samples **0**–**9** generally exhibited lower antioxidant capacity. In this study, TEAC values obtained from the ABTS assay ranged from 0.098 to 0.207, while those from the DPPH assay ranged from 0.080 to 0.216. The TEAC values from both assays are generally comparable, reflecting a relatively consistent ranking of antioxidant potency among the samples. Results from both methods identified samples **5** and **6** as the most potent, whereas samples **0** and **1** exhibited the lowest antioxidant activity. Additionally, samples **4**, **7**, and **8** showed relatively high activity in both assays. Samples **6** and **7** showed the highest total phenolic content (TPC).

### 2.2. Urolithins and Phenolic Acid Analysis

Quantification of six phenolic compounds [3,8-Dihydroxy-6*H*-dibenzo[*b,d*]pyran-6-one (**urolithin A-Uro**-**A**), 3-Hydroxy-6*H*-dibenzo[*b,d*]pyran-6-one (**urolithin B**-**Uro-B**), caffeic acid (**CA**), ferulic acid (**FA**), *trans*-4-Hydroxycinnamic acid (**HCA**), and rosmarinic acid (**RA**)] across the ten shilajit samples **0**–**9** revealed substantial variation in both the total phenolic content and the detectability of individual analytes, with a consistent relationship to sample origin (Table 2).

Samples **6** (40% Carpathian + 60% Sierra Madre) and **7** (30% Carpathian + 70% Sierra Madre) showed the greatest enrichment in nearly every analyte measured (Table 2). Hydroxycinnamic acid (**HCA**) reached its two highest values in these samples (181.60 and 240.71 mg kg^−1^, respectively), several-fold higher than in any single-origin sample, including pure Carpathian material (28.40 mg kg^−1^) or the Sierra Madre-containing but non-Carpathian blend (16.90 mg kg^−1^). A similar pattern was observed for **Uro-A** (27.09 and 34.10 mg kg^−1^, versus 3.20–18.17 mg kg^−1^ in all other samples), caffeic acid (37.05 and 16.42 mg kg^−1^), and ferulic acid (91.48 and 64.80 mg kg^−1^). Most notably, **Uro-B** and rosmarinic acid -both below the limit of detection (LoD) in the majority of matrices-were quantifiable almost exclusively in samples **6** and **7**. Notably, this pattern mirrors the selenium enrichment previously observed in the same two samples (Table 1), raising the possibility of a shared underlying driver-for example, a common botanical or microbial source of the humic fraction at these deposits that concentrates both selenium and polyphenolic constituents simultaneously. Ferulic acid displayed a more binary pattern than the other analytes: it was undetectable (<LoD) in the pure Andean sample (**0**) and the 30% Mongolian/70% Himalayan blend (**2**), yet readily quantifiable (16.08–91.48 mg kg^−1^) in every sample containing a Sierra Madre, 50–level Mongolian, or Carpathian component. This suggests that ferulic acid, or its bound precursors, may be differentially associated with specific botanical inputs to the humification process at certain deposit sites, and could serve as a useful qualitative marker for excluding Andean or high-Himalayan-fraction origins during authentication testing. **Uro-A** was detected in all ten samples (3.20–34.10 mg kg^−1^), a finding consistent with a recent HPLC-based survey that reported **Uro-A** at approximately 0.03–0.42% in raw shilajit, extracts, and resin, and proposed it as a consistent chemical marker across sources [36,37].

Taken together, the phenolic and urolithin data reinforce a pattern established by the elemental analysis: the Carpathian/Sierra Madre combination (**6**, **7**) is compositionally distinct from all other origins and blends examined, showing simultaneous enrichment in selenium, **HCA**, **Uro-A** and **Uro-B**, **CA**, **FA**, and **RA**. This convergence across independent analytical domains (inorganic and organic) strengthens the case that this specific origin combination merits targeted follow-up, both to confirm reproducibility with additional independently sourced material and to explore its underlying botanical or microbiological basis.

### 2.3. Elemental Composition of Samples

The elemental profile obtained for the ten shilajit samples analyzed in this study is broadly consistent with previously reported mineral compositions of shilajit from different geographical origins, while also revealing sample-to-sample variability that is characteristic of this geologically derived, plant-humus material (Table 3).

Across all ten samples, calcium and potassium were the dominant elements, with potassium reaching values as high as 61,450 mg kg^−1^ and calcium as high as 19,760 mg kg^−1^. A physicochemical characterization of Himalayan shilajit similarly identified potassium and calcium, alongside magnesium, as the principal minerals, and reported a slightly alkaline pH consistent with a mineral-rich humic matrix [38]. An ED-XRF-based comparison of Afghan and Pakistani shilajit reported calcium and potassium together accounting for well over a third of the inorganic mass in some samples, underscoring that these two elements are a structural hallmark of shilajit rather than an artifact of a single production batch or region [39].

Magnesium followed a similar, though less pronounced, trend, ranging from roughly 3950 to 11,030 mg kg^−1^. Magnesium, potassium, and calcium are frequently discussed together in the shilajit literature because, once complexed with fulvic acid, they are thought to remain more readily bioavailable; fulvic acid’s chelating capacity is proposed to enhance the transport of these minerals across cell membranes, which has been used to rationalize shilajit’s traditional use in supporting electrolyte balance and mitochondrial energy metabolism [38].

Sodium, aluminum, and iron showed intermediate concentrations but considerably greater relative variability among samples than the three major elements. Aluminum in particular ranged from about 35 mg kg^−1^ to nearly 750 mg kg^−1^, an almost 20-fold difference across the sample set, while iron ranged from roughly 120 to 825 mg kg^−1^. Aluminum and iron are both known to derive largely from the silicate and oxide mineral matrix associated with the parent rock rather than from the organic (humic/fulvic) fraction of shilajit, and their concentration has been shown to vary considerably with the specific rock formation and mountain region from which a given shilajit deposit is sourced. The wide range observed here is therefore plausibly attributable to differences in the geological source material of the individual samples rather than to analytical inconsistency, a conclusion also drawn in a comparative ICP-MS survey of shilajit samples from multiple regions, which reported pronounced inter-sample variation in minor and trace elements even when major elements remained comparatively stable [40].

Trace elements of nutritional relevance such as zinc, copper, and selenium were present at much lower concentrations (single- to low double-digit mg kg^−1^), consistent with their classification as trace, rather than major, constituents of shilajit. These three elements are of particular interest because of their established physiological roles: zinc is essential for immune modulation and wound healing, copper participates in redox enzyme systems, and selenium is a cofactor for antioxidant enzymes such as glutathione peroxidase. Sample **6** stood out with a selenium concentration (4.27 mg kg^−1^) and zinc concentration (35.16 mg kg^−1^) considerably above the rest of the set, which may again reflect localized geochemical enrichment rather than a processing artifact, though this cannot be confirmed without additional geological provenance data.

Potentially toxic elements chromium, cadmium, and lead were detected in all samples at low concentrations, and their levels warrant comparison with internationally recognized safety thresholds for herbal and traditional medicinal products. The FAO/WHO maximum permissible limits commonly cited for herbal medicines are 0.3 mg kg^−1^ for cadmium and 10 mg kg^−1^ for lead [41]. Measured cadmium concentrations in the present samples (0.02–0.07 mg kg^−1^) remained well below this threshold, and lead concentrations (0.10–2.18 mg kg^−1^) likewise stayed far below the 10 mg kg^−1^ limit in every sample. Chromium, for which the FAO/WHO has historically not set a formal maximum permissible limit for medicinal herbs, nonetheless remained low in absolute terms (0.31–2.95 mg kg^−1^) relative to values reported as concerning in other herbal matrices, where a commonly cited evaluation standard for chromium in herbal medicines is around 2 mg kg^−1^. It is worth noting that regulatory limits for heavy metals in herbal products are not fully harmonized internationally; for example, the Chinese Pharmacopoeia revised its cadmium limit from 0.3 to 1.0 mg kg^−1^ in 2020, while Russian and European standards for lead and cadmium also differ from WHO recommendations [42].

The elemental data shows clear associations between origin and mineral profile, even though several samples represent blends rather than single-source material. Potassium is the clearest discriminator. Pure Andean shilajit (sample **0**) and all Himalayan-containing blends (samples **1**–**3**) show markedly elevated potassium (57,300–61,450 mg kg^−1^), while samples without a Himalayan component-Sierra Madre/Mongolian (**4**), Carpathian (**5**), Carpathian/Sierra Madre blends (**6**–**7**), Mongolian (**8**), and Carpathian/Mongolian (**9**)—cluster substantially lower (27,500–38,300 mg kg^−1^). This suggests that Himalayan and Andean parent rock are comparatively potassium-rich relative to the Carpathian, Sierra Madre, and Mongolian sources.

Aluminum and iron follow a related pattern: both are highest in the Andean sample and the Himalayan/Mongolian blend (Al 428–750 mg kg^−1^, Fe 786–825 mg kg^−1^), consistent with a more aluminosilicate- and iron-oxide-rich mineral matrix at these locations, whereas Carpathian-, Sierra Madre-, and Mongolian-dominant samples show Al and Fe an order of magnitude lower. This points to differences in the underlying geological substrate (e.g., volcanic/metamorphic versus more calcareous or humic-dominant deposits) rather than to processing differences, since the trend tracks the origin blend consistently across multiple samples. Selenium shows a distinct signature tied to the Carpathian–Sierra Madre combination: samples **6** and **7** (both Carpathian/Sierra Madre blends) have the highest selenium values (3.3–4.3 mg kg^−1^) in the entire set, well above pure Carpathian (sample **5**, 0.16 mg kg^−1^) or Sierra Madre-containing non-Carpathian blends. This implies a possible synergistic or source-specific selenium enrichment linked specifically to that combination rather than to either source alone.

Magnesium is lowest in samples **8** and **9**, suggesting a comparatively magnesium-poor substrate at that origin, while cadmium and chromium remain low and relatively uniform across all origins, indicating that these two elements are less sensitive geographic markers in this dataset. The concentrations of As (0.196, 0.058, and 0.038 µg g^−1^ in 2016, 2019, and 2022, respectively), Cd (0.593, 0.295, and 0.208 µg g^−1^ in 2016, 2019, and 2022, respectively), and Pb (0.06, 0.044, and 0.05 µg g^−1^ in 2016, 2019, and 2022, respectively) are found in dark chocolate [43]. This comparison is descriptive and is not intended as a safety benchmark, since dark chocolate is itself a recognized dietary source of cadmium and lead.

### 2.4. Acute Toxicity, Clinical Signs, Food Intake, Water Intake, and Body Weight During the 14-Day Observation Period

Daily clinical observations throughout the 14-day monitoring period revealed no mortality or overt clinical signs of toxicity across all ten treatment groups (2000 mg kg^−1^) and the control group. Specifically, no treatment-related abnormalities were observed regarding skin and fur appearance, ocular condition, salivation, or respiratory patterns. Furthermore, urine color, fecal consistency, and somatomotor activity remained normal, with no behavioral alterations or lethargy detected in any of the animals.

**Figure 1:**
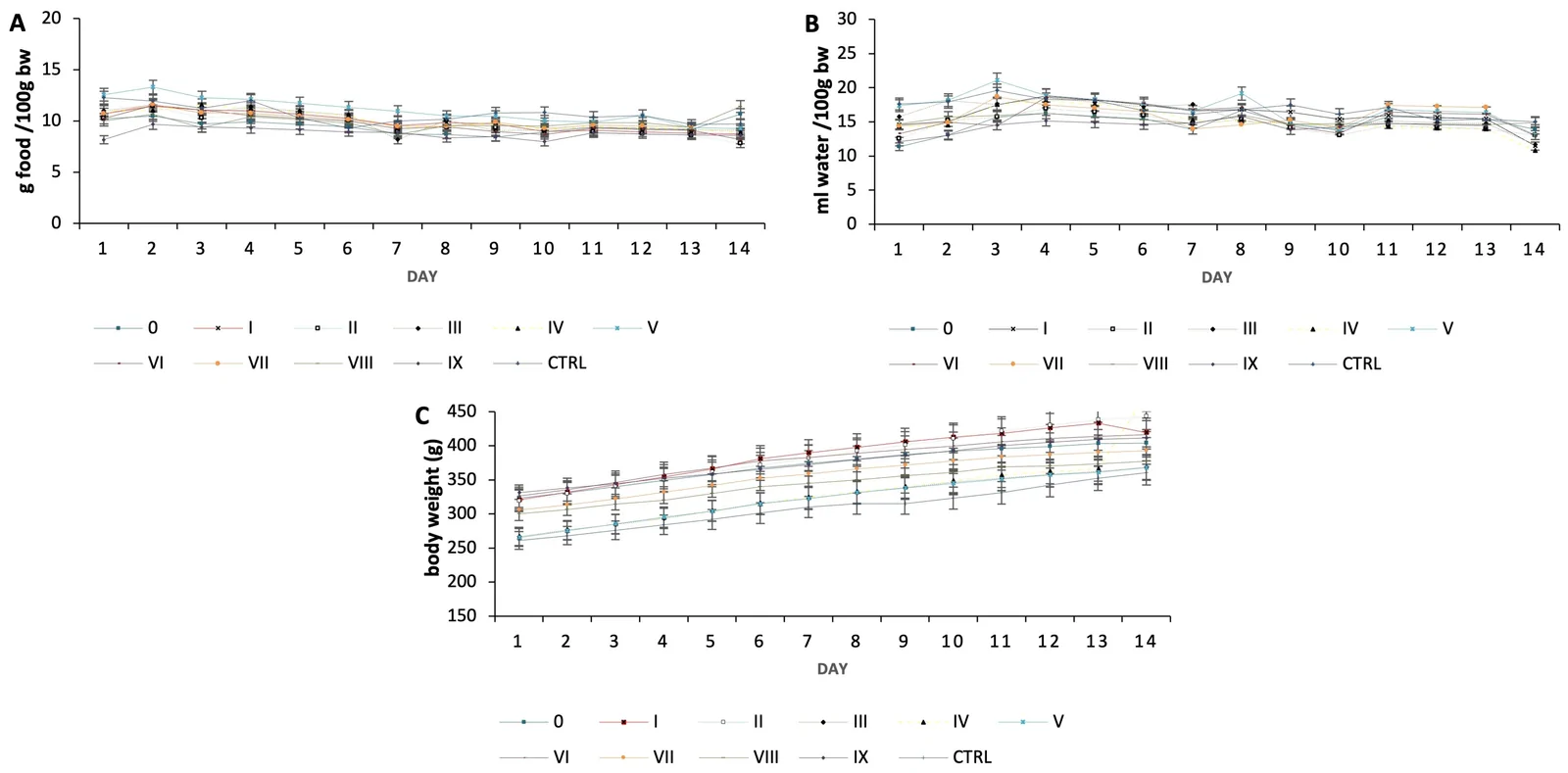
Food intake (**A**), water intake (**B**), and body weight (**C**) in rats.

Changes in food intake (Figure 1A), water intake (Figure 1B), and body weight (Figure 1C) were monitored over a 14-day period in rats treated with different test substances, in accordance with OECD Guideline 423 [44]. Food intake, expressed as g/100 g body weight, showed minor day-to-day fluctuations throughout the experimental period in all treated samples as well as in the control sample (Figure 1A). Slightly higher values were observed during the initial days, followed by stabilization during the remainder of the study. Throughout the 14-day observation period, food consumption in all treated samples remained comparable to that of the control sample, with no consistent trend towards a reduction or progressive alteration. A similar pattern was observed for water intake (Figure 1B). Water consumption exhibited moderate daily variations, with a slight increase during the early phase of the experiment and relatively stable values during the later days. No consistent increase or decrease in water intake was observed in any treated sample compared with the control sample over time. Moreover, body weight measurements (Figure 1C) suggested a continuous and progressive increase throughout the entire experimental period in all samples. Body weight gain followed an approximately linear, time-dependent pattern, without abrupt decreases or growth retardation. All treated samples showed body weight profiles comparable to the control sample, indicating preserved general health status and normal growth during the study.

Monitoring of food intake, water intake, and body weight represents essential indicators of systemic toxicity in studies conducted according to OECD Guideline 423 [44]. In the present study, none of these parameters showed clinically relevant or progressive alterations during the 14-day observation period. The minor fluctuations in food and water intake observed over time were also present in the control sample and are therefore considered to reflect normal physiological variability rather than treatment-related effects. The absence of a sustained decrease in food or water consumption suggests that administration of the tested compounds did not adversely affect appetite, hydration status, or overall well-being of the animals [45]. Moreover, the consistent increase in body weight across all experimental samples further supports the lack of systemic toxicity. Body weight loss or impaired weight gain is regarded as a sensitive indicator of adverse effects in acute and subacute toxicity studies [46]. However, no such effects were detected in this study. On the contrary, animals exhibited steady weight gain consistent with normal physiological growth. Overall, these findings indicate that oral administration of the compounds at the tested dose does not induce adverse effects on food intake, water intake, or body weight during the 14-day observation period. These results are in agreement with the criteria outlined in OECD Guideline 423 and support the conclusion that the compounds do not produce overt signs of acute oral toxicity under the conditions of this study.

### 2.5. Histopathological Analysis of Tissues After Oral Application of Shilajit

In order to provide further insight into the effects of shilajit samples, histological analysis of the liver and kidney was performed to assess potential treatment-related structural alterations. Histological examination revealed the presence of mild to moderate hepatic changes across experimental samples **0**–**9** (Figures S1–S22). Importantly, the type and extent of histological alterations varied among samples (Table S3). Sinusoidal dilatation was absent in the control group and in sample **1**, and was graded minimal to mild (1–2) in the remaining samples. Hydropic degeneration of hepatocytes was absent in the control group and present in all treated samples, ranging from minimal to moderate (1–3), with the highest grade recorded in sample **6**. Ballooning degeneration was additionally detected in samples **1**, **2**, and **7**, indicating a higher degree of hepatocellular injury in these samples (Figures S2, S3 and S8). Additionally, necrotic alterations were mainly focal and were present in samples **0**, **1–7**, and **9**. Minimal confluent necrosis, representing more extensive hepatocellular damage, was observed only in samples **2** and **7** (Figures S3 and S8). Inflammatory responses were identified in specific samples. Minimal to mild lymphocyte infiltration was detected in samples **3**, **5**, **6**, **8**, and **9**, while Kupffer cell hyperplasia was observed in samples **1** and **9**, suggesting activation of hepatic immune and phagocytic cells in these animals. No fibrotic changes were detected in any of the examined liver samples, indicating the absence of irreversible hepatic injury during the study period (Table S3).

Moreover, examination of the kidney revealed the presence of degenerative, necrotic, and tubular alterations across experimental samples **0**–**9** (Figures S12–S22). The most frequently observed changes included hydropic degeneration, desquamation, necrosis, and the presence of eosinophilic intratubular material. Tubular necrosis was detected in all treated samples (grades 1–3), and hydropic degeneration in all treated samples except sample **3** (grades 1–3), indicating varying degrees of tubular epithelial injury. Different levels of atrophy and desquamation were observed in all experimental samples, while eosinophilic intratubular material was present in samples **0**, **2**, and **6–9**. Glomerular atrophy was detected exclusively in sample **2**, suggesting a localized glomerular alteration in this sample. Congestion was observed in the control and in samples **3** and **4**, while mild interstitial edema was recorded only in sample **5**. No interstitial hemorrhage or interstitial inflammatory infiltrate was observed in any sample. Alterations of vascular walls were recorded only in sample **4** (grade 2). Overall, the histopathological findings showed degenerative and necrotic alterations of variable type and severity in hepatic and renal tissues. Hepatic changes were predominantly degenerative, with focal necrosis observed in several samples and confluent necrosis in samples **2** and **7**, while no hepatic fibrosis was observed. Renal alterations were predominantly tubular, with no pronounced interstitial lesions identified. In conclusion, the absence of pronounced interstitial inflammation or hemorrhage suggests that the observed renal alterations were limited in severity and predominantly confined to tubular compartments.

### 2.6. Testosterone, Gonadotropins, and Lipid Level Investigation

Serum total cholesterol levels showed no statistically significant differences across the experimental samples (**0**–**9**) compared to the control sample (2.23 ± 0.20 mmol L^−1^). Values ranged from 1.93 ± 0.39 mmol L^−1^ (**6**) to 2.63 ± 0.25 mmol L^−1^ (**8**), with most samples exhibiting levels comparable to or slightly above control (Table 4). Serum triglyceride concentrations were numerically lower than control (1.27 ± 0.21 mmol L^−1^) in most treated groups, with the lowest values in sample **8** (0.48 ± 0.04 mmol L^−1^) and sample **6** (0.70 ± 0.22 mmol L^−1^). None of these differences reached statistical significance. High-density lipoprotein (HDL) levels were elevated in several experimental samples relative to controls (0.66 ± 0.11 mmol L^−1^). Significant increases were observed in sample **0** (0.91 ± 0.05 mmol L^−1^, *p* < 0.05) and sample **1** (0.92 ± 0.10 mmol L^−1^, *p* < 0.05). Other samples displayed moderate elevations (e.g., samples **8** and **9**), while sample **6** remained close to control values. Low-density lipoprotein cholesterol (LDL) concentrations varied without a consistent pattern across samples, ranging from 0.60 ± 0.27 mmol L^−1^ (sample **7**) to 1.48 ± 0.36 mmol L^−1^ (sample **8**), with no significant differences compared to control (0.92 ± 0.18 mmol L^−1^). The atherogenic index (AI) was reduced in most treated samples compared to the control (1.39 ± 0.09). Notable numerical improvements occurred in samples **0** (0.86 ± 0.20, *p* < 0.05), **7** (0.77 ± 0.31), and **3** (0.94 ± 0.44), indicating a shift toward a less atherogenic lipid profile, although significance was limited to sample **0** in this parameter. The ERF value (control: 3.39 ± 0.37) was lower in nearly all experimental samples (range 2.64–3.01), with the lowest values in samples **0** (2.64 ± 0.16) and **7** (2.70 ± 0.15). Free testosterone concentrations were markedly and significantly elevated in samples **6** (18.16 ± 1.34 pg mL^−1^, *p* < 0.01) and **7** (20.69 ± 2.57 pg mL^−1^, *p* < 0.001) compared to control (5.86 ± 0.79 pg mL^−1^), representing approximately up to 3.5-fold increases after two weeks of treatment. Other samples showed variable responses, with mild elevations in samples **1** and **2** and a notable reduction in sample **8** (2.71 ± 1.04 pg mL^−1^).

The present study indicates that the tested interventions exerted differential effects on lipid metabolism and androgen status, with several samples showing improvements in key cardiovascular risk markers despite modest changes in total cholesterol and LDL. The most favorable lipid profile alterations were observed in samples exhibiting elevated HDL (particularly samples **0** and **1**) and reduced triglycerides (notably sample **6**), resulting in lower AI values.

Samples **6** and **7** were not pre-specified for endocrine follow-up. They were selected post hoc, on the basis of the free testosterone results of the full ten-sample screen (Table 4), in which these two samples showed the highest concentrations. Total testosterone and the gonadotropins LH and FSH were subsequently measured in these two samples only (Table 5). This analysis is therefore exploratory and hypothesis-generating rather than confirmatory. Both samples showed approximately twofold higher total testosterone than control, while LH was similar across groups (≈0.45 IU L^−1^), and FSH remained below the LoD in all groups.

A rise in testosterone without a concomitant rise in gonadotropins would be consistent with androgen-mediated negative feedback on the hypothalamic–pituitary–gonadal (HPG) axis, but the present data cannot suggest this: FSH was below the limit of detection in all groups, including the control, and the LH comparison was severely underpowered. The present data are insufficient to establish strong conclusions about the mechanism responsible for the testosterone increase. Consistent with our results, higher testosterone levels accompanied by low or unchanged LH and FSH levels have been documented in male BALB/c mice treated with biotin [47], then in rats administered a hydroalcoholic extract of *Eucalyptus* leaves [48], and in Sprague–Dawley rats receiving a formulation containing *Mucuna pruriens*, *Tribulus terrestris*, and *Withania somnifera* [49].

One-way ANOVA indicated significant group effects for HDL, AI, ERF, and free testosterone, but not for total cholesterol, triglycerides, or LDL. Dunnett’s post hoc test was then used to identify which treatment groups differed from the control group. For HDL, significantly higher values were found in samples **0** and **1**; for AI, in sample **0**; and for ERF, in samples **0**, **1**, **3**, **7**, and **8**. For free testosterone, significantly higher values relative to control were found in samples **6** (*p* < 0.01) and **7** (*p* < 0.001). The present findings suggest that treatment with samples **6** and **7** significantly elevated both total and free testosterone concentrations relative to control animals. For total testosterone, both treatment groups showed a significant increase compared to control (sample **6**, *p* < 0.001; sample **7**, *p* = 0.036, Welch-corrected). Although both treatments produced significant increases in testosterone, the effect was more consistent for sample **6**, which showed a lower *p*-value and smaller within-group variability (SD = 1.10 for total testosterone) compared to sample **7** (SD = 1.66).

Post hoc power analysis based on the observed effect sizes indicated high achieved power for total testosterone (control vs. sample **6**: power = 1.0, d = 7.54; control vs. sample **7**: power = 0.8, d = 3.22) and for free testosterone (control vs. sample **6**: power = 1.0, d = 10.07; control vs. sample **7**: power = 1.0, d = 7.16), although such an analysis, being derived from the observed effects, cannot compensate for the small sample size (n = 3). In contrast, power for the LH comparisons was very low (control vs. sample **6**: power = 0.07, d = 0.40; control vs. sample **7**: power = 0.05, d = 0.20), indicating that the study was substantially underpowered to detect a small-to-moderate effect on LH, if one exists. The non-significant LH findings should therefore be interpreted with caution, and larger sample sizes would be required to draw definitive conclusions regarding LH involvement in this model.

## 3. Discussion

The present study provides a comprehensive evaluation of the acute oral toxicity, histopathological impact, serum biochemical profile, and antioxidant capacity of ten distinct samples of shilajit administered at a high single dose (2000 mg kg^−1^ final dried shilajit sample) to male Wistar rats. Across all six analytes, samples **6** (40% Carpathian/60% Sierra Madre) and **7** (30% Carpathian/70% Sierra Madre) consistently exhibited the highest, or among the highest concentrations, and were the only two samples in which all six compounds were simultaneously quantified above the LoD. In contrast, the pure Andean sample (**0**) and the 50% Carpathian/50% Mongolian blend (**9**) generally showed the lowest phenolic content, with three of six analytes (**Uro-B** and **RA** in both samples, plus **FA** in sample **0**) falling below the LoD. Samples derived from Himalayan and Mongolian sources without a Carpathian/Sierra Madre component showed intermediate and more variable phenolic profiles, with no single analyte consistently dominant across this subset.

Overall, the results indicate that shilajit samples tested in this study exhibit an acute oral tolerability under the specific experimental conditions, as evidenced by the absence of mortality, clinical signs of toxicity, and significant alterations in food intake, water consumption, or body weight gain during the 14-day observation period. These findings are consistent with previous reports suggesting acute oral tolerability under the specific experimental conditions for properly processed shilajit. Importantly, the presence of histopathological alterations does not necessarily imply overt systemic toxicity, as biologically active nutritional and nutraceutical compounds may also produce tissue effects at high exposure levels [50]. Nevertheless, histopathological analysis revealed mild to moderate degenerative and necrotic changes in both liver and kidney tissues across most experimental samples. Although no hepatic fibrosis, significant renal interstitial inflammation, or vascular injury was observed, the absence of a recovery group precludes conclusions regarding the persistence or reversibility of these alterations. While these alterations were generally limited in severity and did not progress to irreversible damage (no fibrosis, significant interstitial inflammation, or vascular injury), their presence underscores the importance of source-specific quality control and purification procedures. The variability in the type and extent of organ changes among samples highlights that not all commercially or traditionally available shilajit preparations are equivalent in terms of potential tissue impact. From a biochemical perspective, the most promising findings were the short-term changes in androgen biomarkers, which require confirmation in adequately powered, repeated-dose studies with a dose–response design. While the exact molecular pathways were not evaluated in the present study, the existing literature suggests that mineral pitch components may interact with hepatic lipid pathways, involving hypothetical mechanisms such as PPAR activation or enhanced reverse cholesterol transport. Direct mechanistic validation in targeted hepatic models is required to confirm whether these pathways mediate the acute lipid shifts observed here. The largest increases in serum free testosterone were recorded in samples **6** (~3.1-fold) and **7** (~3.5-fold). The rise in testosterone occurred without a change in LH, while FSH was below the limit of detection in all groups and could not be evaluated. It has been reported in the literature that a 90-day trial of standardized purified shilajit resulted in significant increases in total and free testosterone, with stable gonadotropin (LH and FSH) levels [29]. The compositional enrichment of samples **6** and **7** suggests that one or more constituents of these preparations may contribute to the observed changes in testosterone; however, the present study does not allow attribution of this effect to individual compounds. It has been proposed in the literature that such effects may involve antioxidant protection of Leydig cells and modulation of steroidogenic enzyme activity [29,51,52,53]. While these results offer notable insight into shilajit’s systemic effects, establishing the precise underlying endocrine mechanisms will require subsequent subchronic studies evaluating full HPG axis parameters, including testicular histology. Long-term efficacy and potential applications must be confirmed in future subchronic studies using targeted disease models. It should be noted that the sample size utilized in this study (n = 3 per group) was strictly guided by OECD Guideline 423 for acute toxicity testing to minimize animal usage in accordance with ethical standards. Consequently, the observed biochemical shifts should be interpreted as preliminary biological trends that require robust validation in subsequent, adequately powered subchronic efficacy trials. In addition, a major limitation of this study is that the tested shilajit samples cannot be precisely reproduced, since their origin is documented only at the mountain/region level, without geographical coordinates, and authentication relied on general criteria. Future research should focus on identifying the specific bioactive compounds responsible for the observed endocrine and lipid effects, elucidating their molecular mechanisms (e.g., androgen receptor signaling or Nrf2 activation), and evaluating chronic administration in disease-specific models (e.g., high-fat diet-induced metabolic syndrome or age-related hypogonadism).

## 4. Materials and Method

### 4.1. Shilajit Preparation

Shilajit samples were not collected as part of a geologically referenced sampling campaign. Consequently, exact coordinates and site-level ecological descriptors (including vegetation inventories around exudation zones) were not available. Therefore, the origin of the samples is reported at the mountain/region level. The shilajit sources evaluated in this study are the Southern Andes of Chile [sampling altitude (s.a.) ≈ 4000 m], the Altai Mountains (s.a. ≈ 3500 m), Nepal (s.a. ≈ 5000 m), the Cerro Mohinora area of Mexico (s.a. ≈ 2500 m), and Ukraine (s.a. ≈ 1100 m). The non-commercial shilajit samples (0.5 kg batch) were stored in dark glass containers at room temperature.

Prior to the purification process, 0.5 kg of each individual-origin sample was lyophilized and subsequently ground in a laboratory mill to ensure a homogeneous extraction process. Ten grams (10 g) of finely ground shilajit rock (sample: **0**—Andean shilajit; **1**—50% Mongolian + 50% Himalayan shilajit; **2**—30% Mongolian + 70% Himalayan shilajit; **3**—40% Sierra Madre + 60% Himalayan shilajit; **4**—50% Sierra Madre + 50% Mongolian shilajit; **5**—Carpathian shilajit; **6**—40% Carpathian + 60% Sierra Madre shilajit; **7**—30% Carpathian + 70% Sierra Madre shilajit; **8**—Mongolian Shilajit; and **9**—50% Carpathian + 50% Mongolian Shilajit) was mixed with 200 mL of ultrapure water (1:20 *w*/*v* ratio) at 70 °C for 24 h. The suspension was cooled and rapidly filtered through activated charcoal, followed by sequential filtration using filters with pore sizes of 50, 25, and 10 μm. The resulting filtrate was mixed with 100 mL of 96% (*v*/*v*) ethanol and vigorously stirred for 24 h. The samples were then centrifuged at 5000 rpm, and the organic solvent in the supernatant was removed under reduced pressure at 45 °C. Finally, the resulting samples were lyophilized. The samples were stored in airtight, light-protected containers (shilajit constituents can be photosensitive) with desiccant at 4 °C. Extraction yields were in the range of 68–74%.

### 4.2. Antioxidant Activity

The samples were evaluated for their antioxidant activity using two assays, ABTS (2,2′-azino-*bis*(3-ethylbenzothiazoline-6-sulfonic acid)) and DPPH (2,2-diphenyl-1-picrylhydrazyl). Sample solutions were prepared by dissolving the samples in phosphate-buffered saline (PBS) at concentrations of 10, 5, 2.5, 1, 0.5, and 0.1 mg mL^−1^. The antioxidant activity of the samples was assessed using the ABTS radical-scavenging assay. The ABTS^•+^ radical cation stock solution was prepared by reacting ABTS (4.912 mL, 7 mM in PBS) with potassium persulfate (0.088 mL, 140 mM in distilled water) in the dark for 16 h. The resulting solution was then diluted with PBS until the absorbance at 734 nm reached 0.700 ± 0.02. For the assay, 20 μL of each sample solution was mixed with 2 mL of the diluted ABTS^•+^ solution, shaken, and incubated in the dark for 10 min. Absorbance of the resulting solution was recorded at 734 nm using a Shimadzu 1700 spectrophotometer (Kyoto, Japan). All experiments were performed in triplicate. The percentage inhibition of ABTS^•+^ was calculated as follows:

ABTS radical Inhibition (%) = (Ac − As)/Ac × 100%

where Ac and As are the absorbances of the control and the sample solutions, respectively. Trolox and ascorbic acid were used as standard antioxidant molecules. The IC_50_ values of the samples and reference compounds were determined, and, further, Trolox Equivalent Antioxidant Capacity (TEAC) was calculated by dividing the IC_50_ value of Trolox with the IC_50_ value of the corresponding samples. Alongside the ABTS assay, the DPPH test was also carried out to assess the antioxidant capacity of the samples. A fresh DPPH solution (0.1 mM in methanol) was prepared. For the assay, 100 μL of each solution was mixed with 900 μL of the DPPH solution, and the mixture was incubated in the dark at room temperature for 30 min, after which the solution absorbance was measured at 517 nm using a Shimadzu 1700 spectrophotometer. All experiments were performed in triplicate.

The percentage inhibition of DPPH was calculated using

DPPH Radical Inhibition (%) = [1 − (As − Ab)/Ac] × 100%

where As is the absorbance of the sample in DPPH solution, Ac is the absorbance of the control (100 μL methanol in 900 μL DPPH solution), and Ab is the absorbance of the sample (100 μL) in methanol. IC_50_ and TEAC were evaluated following the same procedure as the previous test. Statistical evaluation was carried out using independent *t*-tests. For all comparisons, differences between the control and corresponding samples were considered statistically significant at *p* < 0.05. All measurements were performed in triplicate, and values are expressed as mean ± standard deviation.

The total phenolic content (TPC) of shilajit samples **0**–**9** was determined using the previously reported the Folin–Ciocalteu colorimetric method [54]. The Folin–Ciocalteu (F–C) reagent was prepared following the published methodology [55]. Each sample (1 g) was extracted with methanol (20 mL, 99.9%) at room temperature for 24 h. The resulting extracts were centrifuged, decanted, and filtered through 0.45 µm nylon syringe filters. An aliquot (250 μL) of each extract was mixed with F–C reagent (five-fold diluted, 2.50 mL) and allowed to stand for 15 min. Freshly prepared Na_2_CO_3_ solution (2.25 mL, 10%, *w*/*v*) was then added, and the mixture was thoroughly vortexed. After 2 h, the absorbance was measured at 765 nm. Validation parameters are presented in Table S1.

### 4.3. LC-MS/MS Analysis

In-house validation of the LC-MS/MS method was conducted according to the guidelines from the International Conference on Harmonization (ICH), Validation of Analytical Procedures: Text and Methodology Q2 [56]. Quantification of **Uro-A** and **Uro-B** [57] and phenolic acid [**CA** (99.3%), **FA** (99.98%), **HCA** (99.84%), and **RA** (99.7%)] contents were determined using a published protocol [58]. Analytes were analyzed in MRM mode **CA** 179 → 135 CE −22 V, and 179 → 106 CE −32 V; **FA** 193 → 134 CE −20 V, and 193 → 178 CE −18 V; **HCA** 163 → 119 CE −24 V, and 163 → 93 CE −44 V; **RA** 359 → 161 CE −24 V, and 359 → 197 CE −26 V, **Uro-A** 227 → 198 CE 35 V; 227 → 154.1 CE 30 V and **Uro-B** 211 → 139 CE 32 V; 211 → 167 CE 33 V.

### 4.4. Elemental Analysis

Concentrations of elements (Na, Mg, Al, K, Ca, Cr, Fe, Cu, Zn, Se, Cd, Hg, As, and Pb) were determined by inductively coupled plasma mass spectrometry (ICP-MS) using an iCap Q mass spectrometer (Thermo Scientific, Bremen, Germany). Quantification was based on the most abundant isotope of each element. The instrument was operated under the following conditions: RF power, 1550 W; cooling gas flow, 14 L min^−1^; nebulizer flow, 1 L min^−1^; collision gas flow, 1 mL min^−1^; and a dwell time of 10 ms [59]. All calibration standards were prepared from starting materials with a purity of 99.999% for each element. Quantitative analysis was performed using a five-point calibration curve (including a zero point) constructed for the following isotopes: ^23^Na, ^24^Mg, ^27^Al, ^39^K, ^44^Ca, ^52^Cr, ^57^Fe, ^63^Cu, ^66^Zn, ^77^Se, ^111^Cd, ^202^Hg, ^75^As and ^206^Pb. All isotopes were measured in KED (Kinetic Energy Discrimination) operating mode to eliminate interferences. A multielement internal standard consisting of ^6^Li, ^45^Sc, ^71^Ga, ^89^Y and ^209^Bi was used in order to correct the instrumental response, improve accuracy, and compensate for possible analyte losses. Validation parameters are presented in Table S2.

### 4.5. Acute Oral Toxicity Evaluation

#### 4.5.1. Ethical Statement

The experimental protocol was approved by the Ethics Committee of the Faculty of Medical Sciences, University of Kragujevac, Serbia (date: 17 December 2025; approval number: 09-12615/3). All experimental procedures in this study were carried out in accordance with Good Laboratory Practice and compliance with the European Directive for the Protection of Vertebrate Animals used for experimental and other scientific purposes (86/609/EES). The experiments were carried out in the Center for Experimental and Preclinical Investigation at the Faculty of Medical Sciences, University of Kragujevac, Serbia.

#### 4.5.2. Animals

Healthy male Wistar albino rats (body weight: 200–250 g; 8–10 weeks) were procured from the Military Medical Academy in Belgrade, Serbia. Animals were housed in stainless steel cages under controlled environmental conditions, including a temperature of 22 ± 2 °C, a 12:12 h light-dark cycle, and a relative humidity of 55–60%. Standard food and water were provided ad libitum. The acute oral toxicity assessment was performed on 33 rats (n = 3 per sample) following the procedures outlined in the Organization for Economic Cooperation and Development (OECD) guideline 423 [44]. The animals were randomly divided into 10 experimental (**0**–**9**) and a control group. The control group received per os a single dose of distilled water. Each experimental sample included rats that received compounds numbered **0**–**9** per os in a single dose of 2000 mg kg^−1^ dissolved in water.

The single oral administration of 2000 mg kg^−1^ was selected as the standardized limit dose in accordance with OECD Guideline 423 (Acute Oral Toxicity—Acute Toxic Class Method) to determine the upper threshold of acute safety LD_50_ > 2000 mg kg^−1^ while minimizing total animal usage.

After oral administration of the test compounds, the rats were housed individually and monitored for 14 days (Figure S23). Food and water intake, as well as body weight, were measured daily. Throughout the observation period, animals were examined for behavioral alterations and clinical signs of toxicity, including changes in fur and skin appearance, ocular condition, salivation, respiratory pattern, urine color, fecal consistency, somatomotor activity, and other deviations from normal behavior [60,61,62].

#### 4.5.3. Histopathological Analysis

After sacrifice, samples of the rat’s liver and kidney were collected and fixed in a 4% paraformaldehyde solution at 4 °C for 24 h. After fixation, tissue sections were dehydrated in increasing concentrations of ethanol, cleared in xylol, and embedded in Histowax^®^ (Histolab Product AB, Göteborg, Sweden). The resulting paraffin blocks were sectioned using a rotary microtome (RM 2125RT, Leica Microsystems, Wetzlar, Germany) to obtain 5 µm thick slices. Tissue sections were stained with hematoxylin–eosin. Microscopic images were obtained using a digital camera (AxioCam ICc1, Carl Zeiss, Oberkohen, Germany) mounted to an Olympus BX51 microscope (AxioScop 40, Carl Zeiss, Oberkohen, Germany). Histopathological evaluation was performed for treated and untreated groups in a blinded manner with respect to treatment allocation. All evaluable liver and kidney sections and microscopic fields were systematically examined, and histopathological alterations were recorded according to their type, distribution, and severity. The micrographs presented in Figures S1–S22 represent selected examples of the histopathological findings identified during the systematic evaluation and were not used as the sole basis for interpretation.

#### 4.5.4. Biochemical Analysis

Biochemical analyses were conducted on serum samples collected after completion of the 14-day protocol (Figure S23). Serum total testosterone was quantitatively determined using a competitive enzyme-linked immunosorbent assay validated for rodent samples (Mouse/Rat Testosterone ELISA, cat. no. TE187S-100, Calbiotech Inc., El Cajon, CA, USA; standard range 0.1–18 ng mL^−1^, analytical sensitivity 0.1 ng mL^−1^). Free testosterone was measured using a species-specific competitive ELISA (Rat Free Testosterone, F-TESTO ELISA Kit, cat. no. CSB-E05097r, Cusabio Technology LLC, Wuhan, China; detection range 0.3–60 pg mL^−1^).

Serum FSH and LH were determined using species-specific sandwich chemiluminescent immunoassay kits (Rat FSH CLIA Kit, cat. no. E-CL-R0255; Rat LH CLIA Kit, cat. no. E-CL-R0024; Elabscience Biotechnology Inc., Wuhan, China). All assays were performed strictly according to the manufacturers’ instructions and measured on an Agilent BioTek Synergy H1 multi-mode microplate reader (Agilent Technologies, Santa Clara, CA, USA). Each sample was assayed in triplicate for every analyte, and all samples for a given analyte were processed within a single run to eliminate inter-assay variability. Intra-assay coefficients of variation were below 10%.

To avoid confounding effects from circadian rhythms, all blood samples were collected post-euthanasia during a standardized morning interval (08:00–10:00 AM). Lipid profile parameters (total cholesterol, triglycerides, HDL, and LDL) were measured spectrophotometrically using a programmed biochemical analyzer and commercial assay kits [63,64].

Atherogenic Index (AI) and Established Risk Factor cardiovascular disease (ERF) are calculated using Equations (1) and (2) [64,65]:

AI = (Total cholesterol − HDL)/HDL(1)

ERF = Total Cholesterol (C)/HDL-Cholesterol (HDL-C)(2)

### 4.6. Statistical Analysis

Data are presented as mean ± standard deviation (SD) unless stated otherwise. Analyses were performed in IBM SPSS Statistics (version 23.0 for Windows).

Antioxidant assays: All measurements were performed in triplicate. IC_50_ and TEAC values for each sample were compared with the corresponding reference standard using independent-samples *t*-tests, with significance set at *p* < 0.05. These data were not included in the ANOVA models described below.

Serum biochemical parameters: The ten treatment groups and the control group were compared across seven parameters total cholesterol, triglycerides, HDL, LDL, AI, ERF and free testosterone—using one-way analysis of variance (ANOVA). Normality within each group was assessed with the Shapiro–Wilk test, and homogeneity of variances with Levene’s test. Where the omnibus ANOVA indicated a significant group effect, each treatment group was compared with the control group using Dunnett’s two-sided *t*-test; no other post hoc procedure was applied to these data. Where the assumption of equal variances was not met, Welch’s correction was applied, and this is indicated in the text.

Gonadotropins and total testosterone: These were measured only in samples **6** and **7** and the control and were compared with the control group as described in Section 2.6; this analysis was exploratory (see Section 2.6).

Food intake, water intake and body weight: Differences among the ten shilajit-treated groups and the control group were evaluated by one-way ANOVA followed by the Bonferroni post hoc test for multiple comparisons.

Post hoc power: Achieved power was computed from the observed group means, pooled standard deviations and group sizes (n = 3 per group) at α = 0.05 (two-tailed) and is reported for the significant findings and for the LH comparisons.

## Figures and Tables

**Table 1:** IC_50_ values of the samples and standard compounds (trolox and ascorbic acid) and corresponding TEAC values and the TPC expressed as milligrams of gallic acid equivalents per gram of shilajit (mg GAE/g shilajit). The results are presented as mean ± SD for *n* = 3.

	ABTS		DPPH		TPC
	IC_50_/mg mL^−1^	TEAC	IC_50_/mg mL^−1^	TEAC	mg GAE/g Shilajit
**0**	3.50 ± 0.29	0.09 ± 0.01	5.55 ± 0.38	0.08 ± 0.01	15.09 ± 3.75
**1**	3.20 ± 0.29	0.10 ± 0.01	6.01 ± 0.26	0.07 ± 0.01	37.72 ± 2.48
**2**	2.17 ± 0.23	0.15 ± 0.02	3.46 ± 0.29	0.12 ± 0.01	45.74 ± 2.18
**3**	2.05 ± 0.26	0.16 ± 0.02	2.70 ± 0.21	0.16 ± 0.01	9.12 ± 0.90
**4**	1.79 ± 0.22	0.19 ± 0.03	2.97 ± 0.22	0.14 ± 0.23	2.40 ± 0.71
**5**	1.65 ± 0.15	0.20 ± 0.02	2.04 ± 0.28	0.21 ± 0.03	11.83 ± 1.74
**6**	1.68 ± 0.15	0.20 ± 0.02	2.46 ± 0.30	0.17 ± 0.02	74.30 ± 5.63
**7**	1.94 ± 0.18	0.17 ± 0.01	3.40 ± 0.33	0.13 ± 0.01	91.59 ± 8.69
**8**	1.92 ± 0.11	0.17 ± 0.01	3.32 ± 0.34	0.13 ± 0.01	5.17 ± 1.40
**9**	2.18 ± 0.24	0.15 ± 0.01	2.72 ± 0.31	0.16 ± 0.02	10.20 ± 3.85
Trolox	0.34 ± 0.01	1 ± 0.006	0.44 ± 0.02	1 ± 0.08	/
Ascorbic acid	0.25 ± 0.01	1.35 ± 0.12	0.10 ± 0.01	4.12 ± 0.48	/

**Table 2:** Content of phenolic compounds from methanol/ethyl acetate extracts in mg kg^−1^ (triplicate, mean ± SD).

	Analyte					
Matrix	Uro-A	Uro-B	CA	FA	HCA	RA
**0**	7.45 ± 1.48	<LoD *	1.48 ± 0.36	<LoD	24.71 ± 2.50	<LoD
**1**	6.40 ± 1.12	<LoD	5.27 ± 1.08	47.11 ± 3.50	3.70 ± 0.94	<LoD
**2**	14.08 ± 3.72	<LoD	4.72 ± 0.90	<LoD	42.16 ± 3.05	<LoD
**3**	5.43 ± 0.91	0.45 ± 0.01	2.40 ± 0.63	19.05 ± 2.83	16.90 ± 1.84	<LoD
**4**	18.17 ± 1.74	0.79 ± 0.01	15.81 ± 2.45	16.08 ± 1.47	85.18 ± 9.15	<LoD
**5**	10.27 ± 2.91	<LoD	12.50 ± 1.36	5.74 ± 1.02	28.40 ± 4.17	<LoD
**6**	27.09 ± 3.47	<LoD	37.05 ± 4.10	91.48 ± 8.30	181.60 ± 15.29	11.83 ± 2.46
**7**	34.10 ± 4.90	<LoD	16.42 ± 1.74	64.80 ± 7.10	240.71 ± 26.80	21.50 ± 1.29
**8**	3.71 ± 0.50	<LoD	11.86 ± 1.09	30.61 ± 5.24	43.96 ± 7.14	0.47 ± 0.19
**9**	3.20 ± 0.64	<LoD	1.45 ± 0.30	24.15 ± 4.31	32.57 ± 5.80	<LoD

* Limit of detection (LoD); LoD (**Uro-B**) = 35 µg kg^−1^; LoD (**CA**) = 15 µg kg^−1^; LoD (**FA**) = 25 µg kg^−1^; LoD (**HCA**) = 15 µg kg^−1^; LoD (**RA**) = 10 µg kg^−1^.

**Table 3:** Elemental composition of samples **0**–**9**. Data are presented in mg kg^−1^ as mean ± SD (n = 3).

	Na	Mg	Al	K	Ca	Cr	Fe	Cu	Zn	Se	Cd	Pb	Hg	As
**0**	614.37 ± 65.03	7962.18 ± 790.68	428.57 ± 49.51	60,784.95 ± 5372.04	13,584.14 ± 1236.67	1.64 ± 0.41	825.17 ± 10.83	6.16 ± 0.82	12.50 ± 3.69	0.91 ± 0.54	0.07 ± 0.01	1.64 ± 0.12	<LoD	0.21 ± 0.05
**1**	715.38 ± 61.19	8740.09 ± 762.85	749.52 ± 76.20	58,406.56 ± 4675.46	19,760.24 ± 2405.76	2.51 ± 0.64	786.30 ± 91.70	3.80 ± 0.52	14.30 ± 1.72	3.15 ± 0.15	0.04 ± 0.01	2.18 ± 0.45	<LoD	0.30 ± 0.05
**2**	1063.93 ± 105.82	11,028.60 ± 1005.24	69.42 ± 9.71	61,450.75 ± 5384.00	14,754.68 ± 1634.75	0.74 ± 0.15	187.41 ± 19.74	4.59 ± 0.46	17.02 ± 1.94	1.41 ± 0.12	0.06 ± 0.00	0.10 ± 0.02	<LoD	0.51 ± 0.09
**3**	453.10 ± 57.26	8416.08 ± 729.63	153.70 ± 21.62	57,301.92 ± 5817.25	11,367.47 ± 1458.95	0.48 ± 0.06	467.21 ± 57.12	1.94 ± 0.70	24.91 ± 2.96	0.25 ± 0.07	0.06 ± 0.00	0.57 ± 0.32	<LoD	0.45 ± 0.05
**4**	721.82 ± 74.11	10,107.40 ± 872.40	34.90 ± 4.21	32,651.64 ± 3624.10	16,739.90 ± 2441.29	2.95 ± 0.27	130.56 ± 23.10	3.97 ± 0.46	12.54 ± 1.74	0.48 ± 0.17	0.03 ± 0.00	0.84 ± 0.02	<LoD	0.96 ± 0.10
**5**	824.98 ± 80.20	10,741.34 ± 1027.02	54.81 ± 5.70	32,108.70 ± 4416.95	15,797.18 ± 1741.53	0.31 ± 0.03	161.95 ± 18.40	2.04 ± 0.63	11.85 ± 1.47	0.16 ± 0.04	0.02 ± 0.00	0.19 ± 0.03	<LoD	2.05 ± 0.18
**6**	847.01 ± 76.47	10,301.47 ± 942.31	38.64 ± 4.78	34,580.09 ± 3351.42	14,751.24 ± 1738.43	1.57 ± 0.08	119.72 ± 13.84	3.97 ± 0.64	35.16 ± 1.28	4.27 ± 0.58	0.02 ± 0.00	0.47 ± 0.27	<LoD	1.41 ± 0.13
**7**	419.73 ± 30.41	4921.70 ± 452.85	90.52 ± 8.24	38,295.96 ± 3647.25	8375.61 ± 907.24	0.82 ± 0.07	274.28 ± 31.70	6.18 ± 0.71	42.10 ± 2.96	3.26 ± 0.41	0.05 ± 0.00	2.18 ± 0.61	<LoD	1.08 ± 0.12
**8**	815.91 ± 75.04	4138.01 ± 393.08	52.47 ± 6.10	27,511.48 ± 3972.28	5792.10 ± 619.57	0.57 ± 0.06	143.82 ± 30.69	7.28 ± 0.64	14.71 ± 1.48	0.21 ± 0.03	0.06 ± 0.00	0.51 ± 0.12	<LoD	2.10 ± 0.38
**9**	368.41 ± 44.90	3951.19 ± 324.42	180.35 ± 13.78	29,187.40 ± 3641.93	15,908.42 ± 1702.80	0.96 ± 0.09	237.10 ± 30.65	1.65 ± 0.50	9.24 ± 1.48	0.32 ± 0.05	0.06 ± 0.00	0.98 ± 0.07	<LoD	1.63 ± 0.26

**Table 4:** Biochemical parameters obtained from rat serum.

Sample	Cholesterol (mmol L^−1^)	Triglycerides (mmol L^−1^)	HDL (mmol L^−1^)	LDL (mmol L^−1^)	AI	ERF	Free Testosterone (pg mL^−1^)
**0**	2.40 ± 0.15	1.43 ± 0.46	0.91 ± 0.05 *	0.77 ± 0.15	0.86 ± 0.20 *	2.64 ± 0.16 **	9.57 ± 1.74
**1**	2.48 ± 0.32	1.27 ± 0.36	0.92 ± 0.10 *	0.92 ± 0.26	0.99 ± 0.24	2.67 ± 0.07 **	10.54 ± 3.50
**2**	2.13 ± 0.08	1.17 ± 0.45	0.74 ± 0.11	0.81 ± 0.23	1.09 ± 0.29	2.93 ± 0.32	11.16 ± 8.51
**3**	2.27 ± 0.44	1.14 ± 0.31	0.81 ± 0.20	0.72 ± 0.27	0.94 ± 0.44	2.81 ± 0.16 *	8.13 ± 0.90
**4**	2.10 ± 0.32	0.92 ± 0.18	0.70 ± 0.09	0.94 ± 0.19	1.35 ± 0.20	3.01 ± 0.16	5.97 ± 2.99
**5**	2.34 ± 0.36	1.08 ± 0.20	0.80 ± 0.14	1.00 ± 0.12	1.25 ± 0.07	2.93 ± 0.07	9.42 ± 2.29
**6**	1.93 ± 0.39	0.70 ± 0.22	0.66 ± 0.17	0.89 ± 0.17	1.38 ± 0.22	2.93 ± 0.25	18.16 ± 1.34 **
**7**	2.06 ± 0.42	1.38 ± 0.26	0.77 ± 0.18	0.60 ± 0.27	0.77 ± 0.31	2.70 ± 0.15 **	20.69 ± 2.57 ***
**8**	2.63 ± 0.25	0.48 ± 0.04	0.91 ± 0.14	1.48 ± 0.36	1.63 ± 0.52	2.89 ± 0.18 *	2.71 ± 1.04
**9**	2.41 ± 0.16	1.24 ± 0.25	0.83 ± 0.10	1.25 ± 0.18	1.49 ± 0.55	2.91 ± 0.19	8.88 ± 1.61
control	2.23 ± 0.20	1.27 ± 0.21	0.66 ± 0.11	0.92 ± 0.18	1.39 ± 0.09	3.39 ± 0.37	5.86 ± 0.79

Note: Values represented mean ± SD, n = 3 rats per sample, and * *p* < 0.05, ** *p* < 0.01, and *** *p* < 0.001 when compared with the control sample (untreated rats).

**Table 5:** Gonadotropins (**LH** and **FSH**) levels obtained from rat serum.

Sample	LH (IU L^−1^)	FSH (ng mL^−1^)	Total Testosterone (ng mL^−1^)
**6**	0.46 ± 0.02	<LoD *	6.45 ± 1.10
**7**	0.45 ± 0.02	<LoD	6.85 ± 1.66
**control**	0.45 ± 0.02	<LoD	2.74 ± 0.69

Note: Values represented mean ± SD, n = 3 rats per sample, * LoD = 0.6 ng mL^−1^.

## Data Availability

The original contributions presented in this study are included in the article/Supplementary Materials. Further inquiries can be directed to the corresponding author.

## Supplementary Materials

The following supporting information can be downloaded at https://www.mdpi.com/article/10.3390/ph19091486/s1. Figures S1–S11: Micrographs of liver tissues 0–9; Figures S12–S22: Micrographs of kidney tissues; Figure S23. Experimental design of the 14-day acute oral toxicity study in rats. Table S1: Calibration parameters; Table S2: Limit of quantification (LoQ) and recovery (%); Table S3: Semi-quantitative grading for liver and kidney tissues (all rats were evaluated); 0 = absent, 1 = minimal, 2 = mild, 3 = moderate, 4 = marked. The control column is the reference against which treatment-relatedness is judged.

## Abbreviations

The following abbreviations are used in this manuscript:

ABTS	2,2′-Azino-bis(3-ethylbenzothiazoline-6-sulfonic acid)
AI	Atherogenic Index
DPPH	2,2-Diphenyl-1-picrylhydrazyl
ECLIA	Electrochemiluminescence Immunoassay
ERF	Established Risk Factor (cardiovascular disease)
GABA	Gamma-Aminobutyric Acid
HDL	High-Density Lipoprotein
IC_50_	Half-Maximal Inhibitory Concentration
LDL	Low-Density Lipoprotein
NAFLD	Non-Alcoholic Fatty Liver Disease
OECD	Organisation for Economic Cooperation and Development
PBS	Phosphate-Buffered Saline
ROS	Reactive Oxygen Species
TEAC	Trolox Equivalent Antioxidant Capacity
WHO	World Health Organization
