# Supplementary material for: Biogeochemical Evaluation of Shilajit: Testosterone-Modulating Effects, In Vivo Toxicity and Lipid Profile Analysis
Source: Pharmaceuticals (Basel). 2026 Sep 17;19(9):1486. doi: 10.3390/ph19091486 (PMC13610426; doi:10.3390/ph19091486)
Supplement: Supplementary file 1 [file pharmaceuticals-19-01486-s001.zip › pharmaceuticals-4531145-supplementary.pdf]

Supplementary Information associated with the paper

Biogeochemical evaluation of shilajit: Testosterone-modulating effects, in vivo toxicity and lipid profile analysis

Nodari Rizun,[1] Vesna Stanković,[2] Jovana Bradić,[3,4] Marijana Anđić,[3,4] Jelena Lađarević,[5] Vladimir Jakovljević,[4,6] Svitlana Zahorodnia,[7] Nenad Janković*[8]

[1]Pürblack Inc., Founder - CEO, Temecula, California, USA

[2]Department of Pathology, Faculty of Medical Sciences, University of Kragujevac, 34000 Kragujevac, Serbia

[3]Department of Pharmacy, Faculty of Medical Sciences, University of Kragujevac, Kragujevac, Serbia

[4]Center of Excellence for Redox Balance Research in Cardiovascular and Metabolic Disorders, 69 Svetozara Markovica St., 34000 Kragujevac, Serbia

[5]Faculty of Technology and Metallurgy, University of Belgrade, Karnegijeva 4, 11000 Belgrade, Serbia

[6]Department of Physiology, Faculty of Medical Sciences, University of Kragujevac, 69 Svetozara Markovica St., 34000 Kragujevac, Serbia

[7]Zabolotny Institute of Microbiology and Virology, The National Academy of Sciences of Ukraine, st. Academician Zabolotny, 154, 03143, Kyiv, Ukraine.

[8]University of Kragujevac, Institute for Information Technologies Kragujevac, Department of Science, Liceja Kneževine Srbije 1A, 34000 Kragujevac, Serbia

*Correspondence: nenad.jankovic@uni.kg.ac.rs

1. **Hystopatologycal analysis of tissues**

1.1. Hystopatologycal analysis of liver tissues

Labels indicate: (a) sinusoidal dilatation; (b1) hydropic degeneration; (b2) ballooning degeneration; (c) focal necrosis; (d) confluent necrosis; (e) Kupffer cell hyperplasia; (f) lymphocyte infiltration; (g) fibrosis.

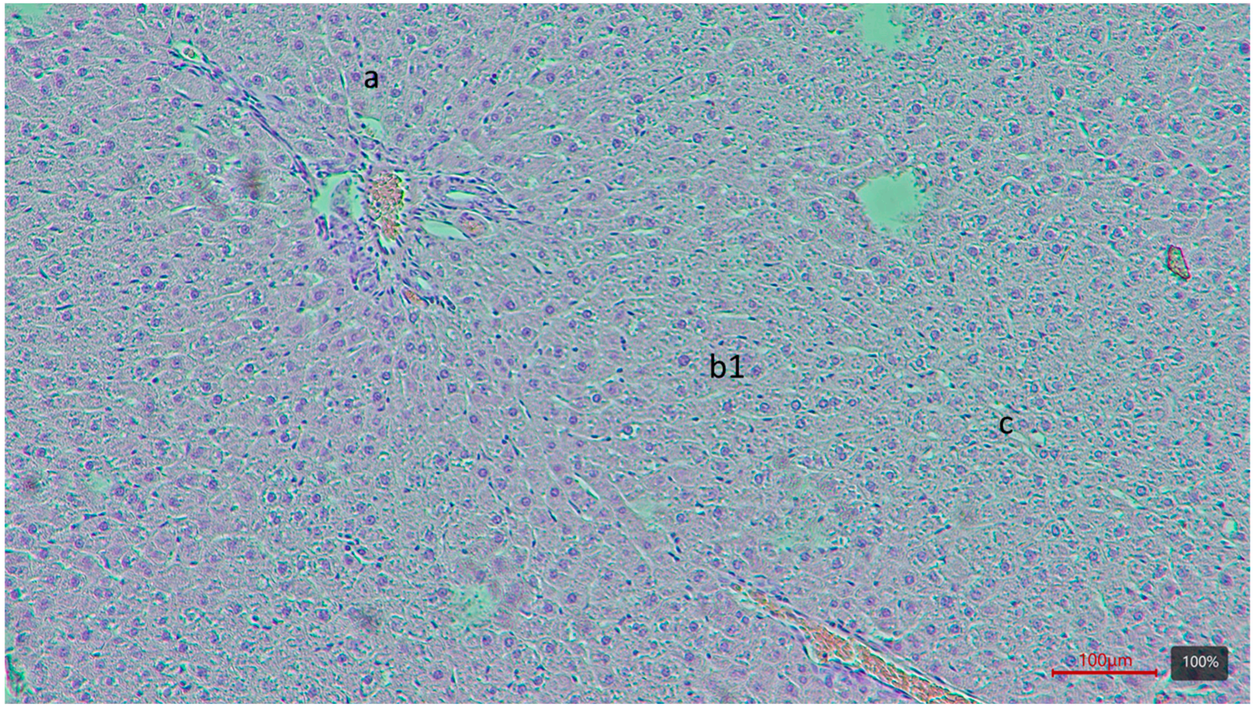

Figure S1. Micrograph of liver tissue sample **0**

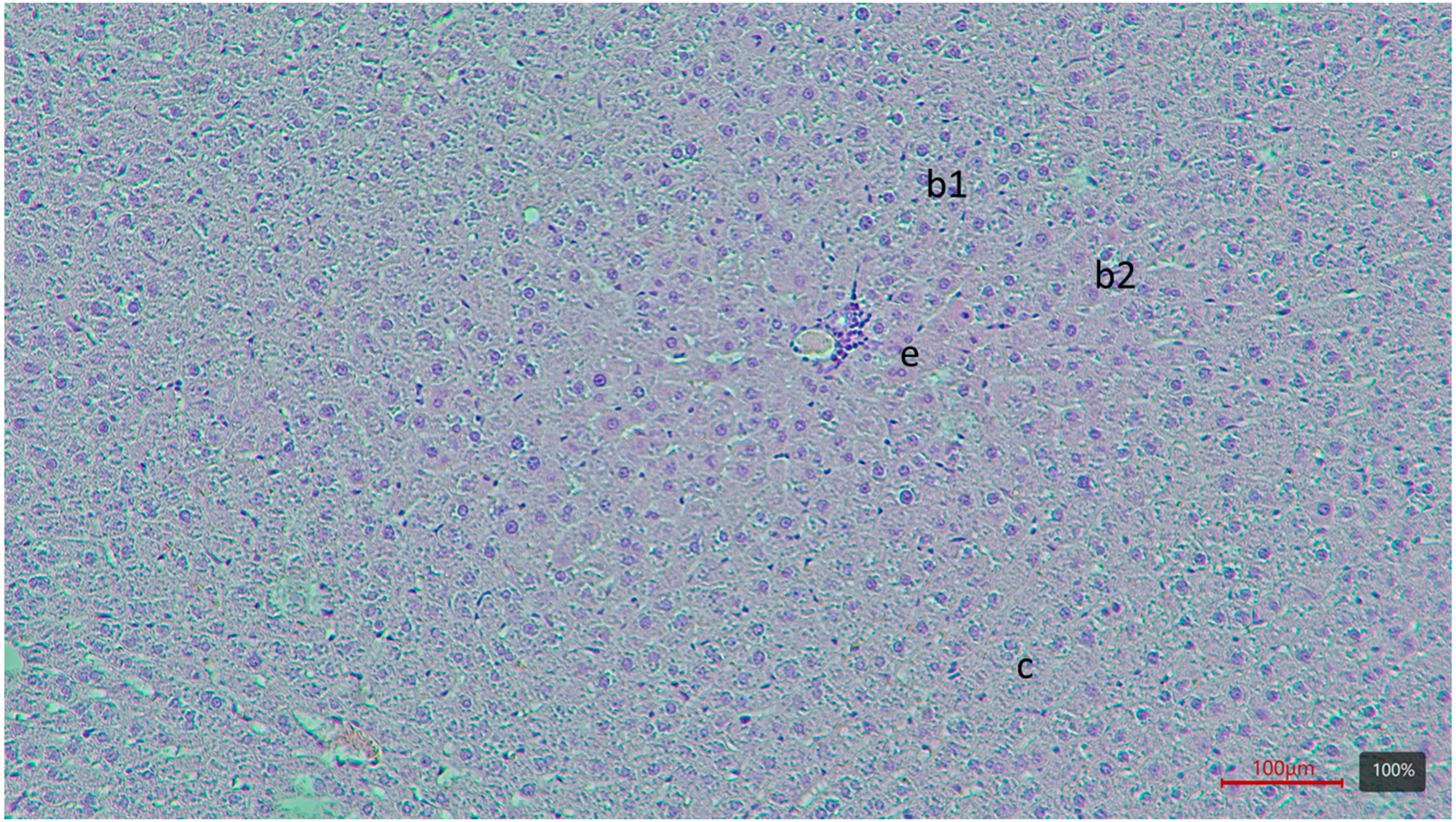

Figure S2. Micrograph of liver tissue sample **1**

,

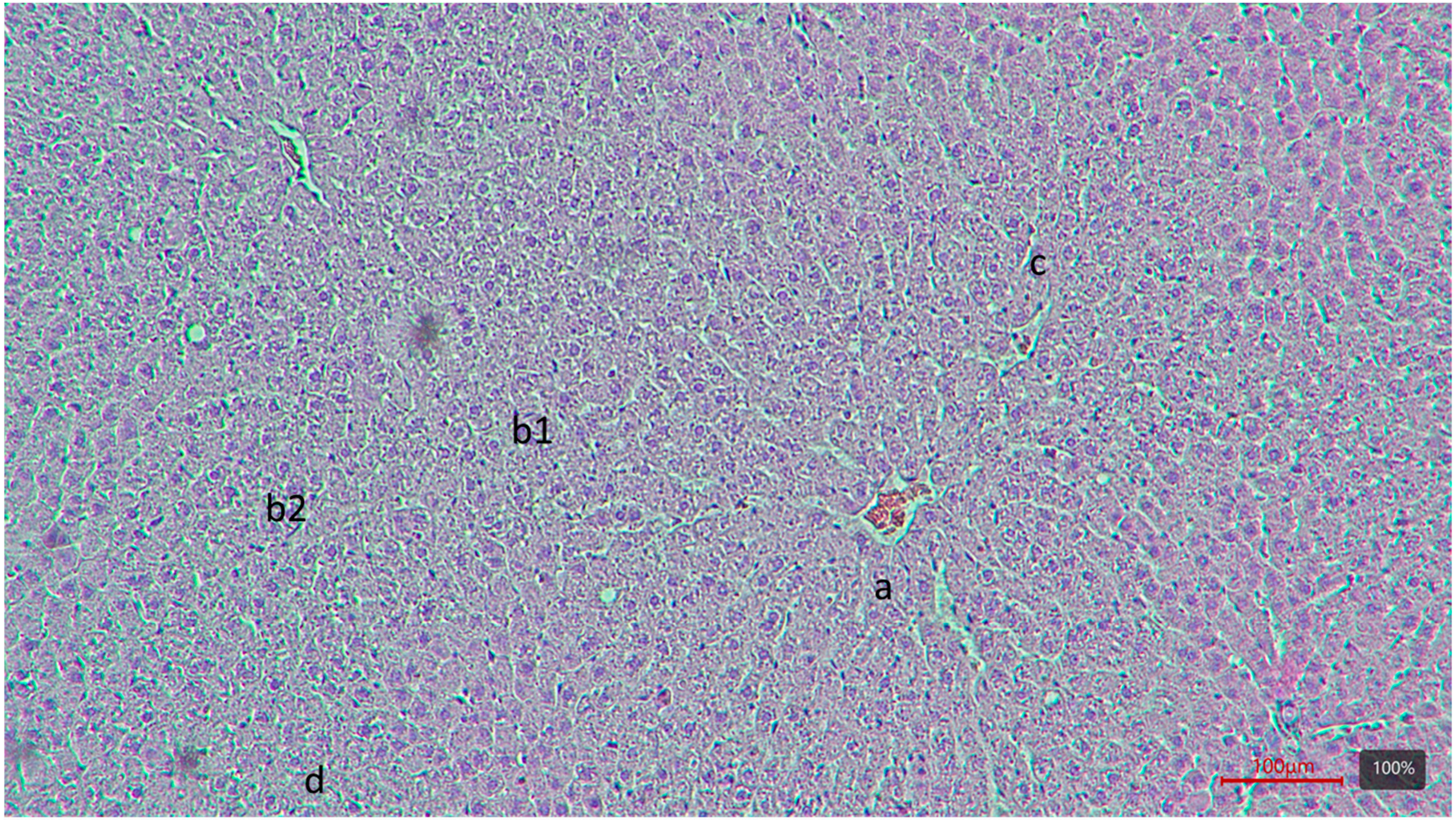

Figure S3. Micrograph of liver tissue sample **2**

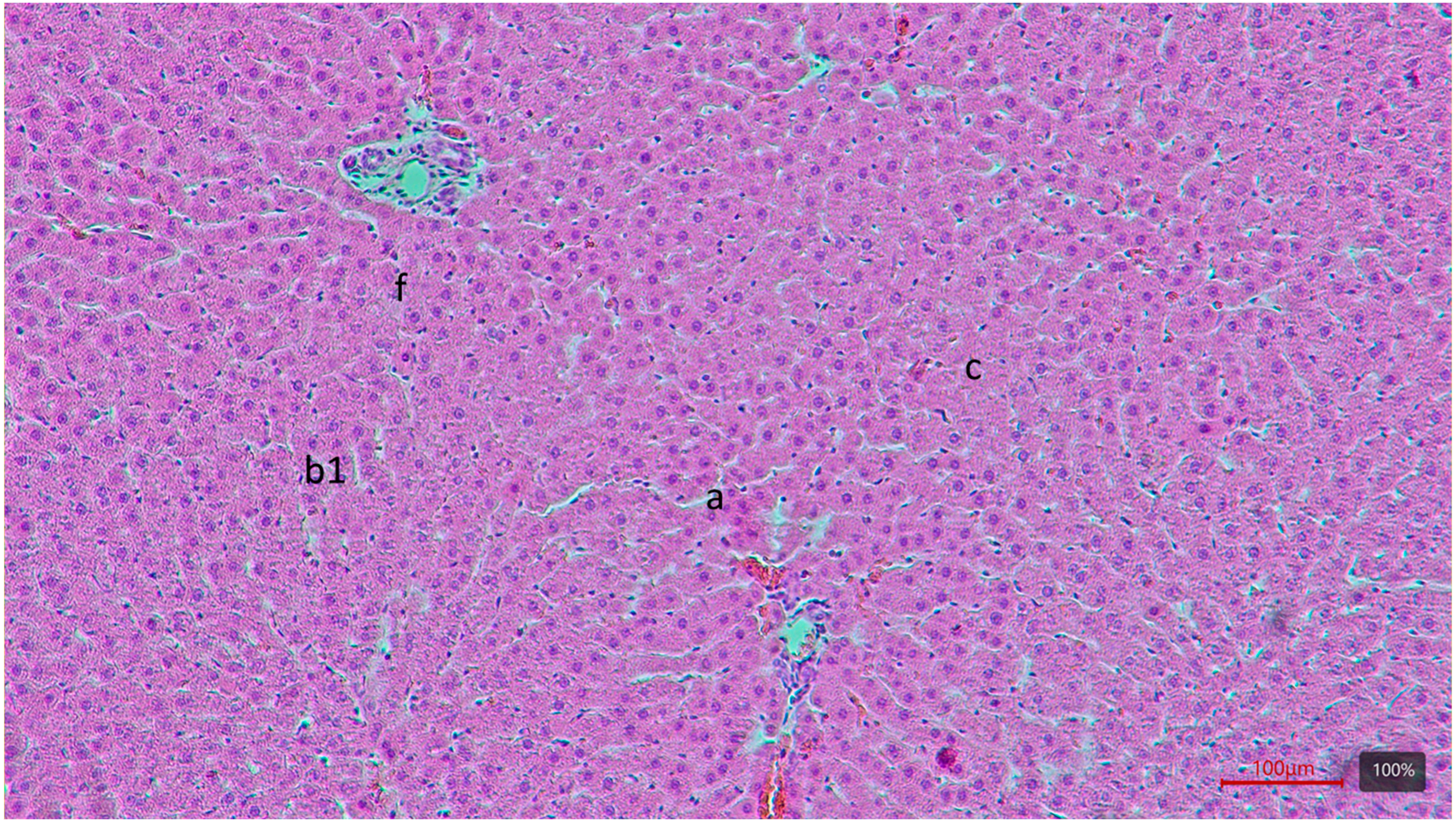

Figure S4. Micrograph of liver tissue sample **3**

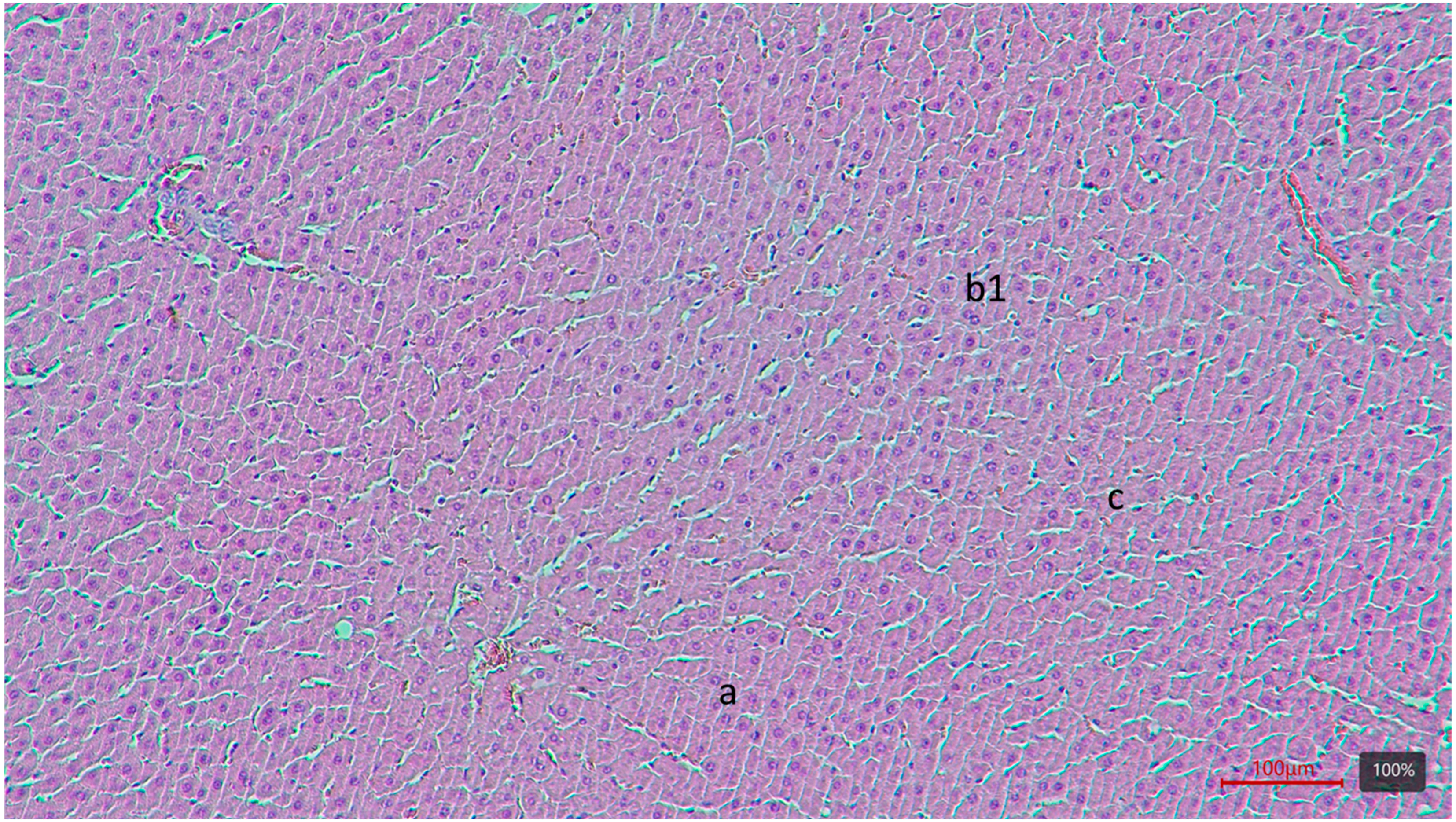

Figure S5. Micrograph of liver tissue sample **4**

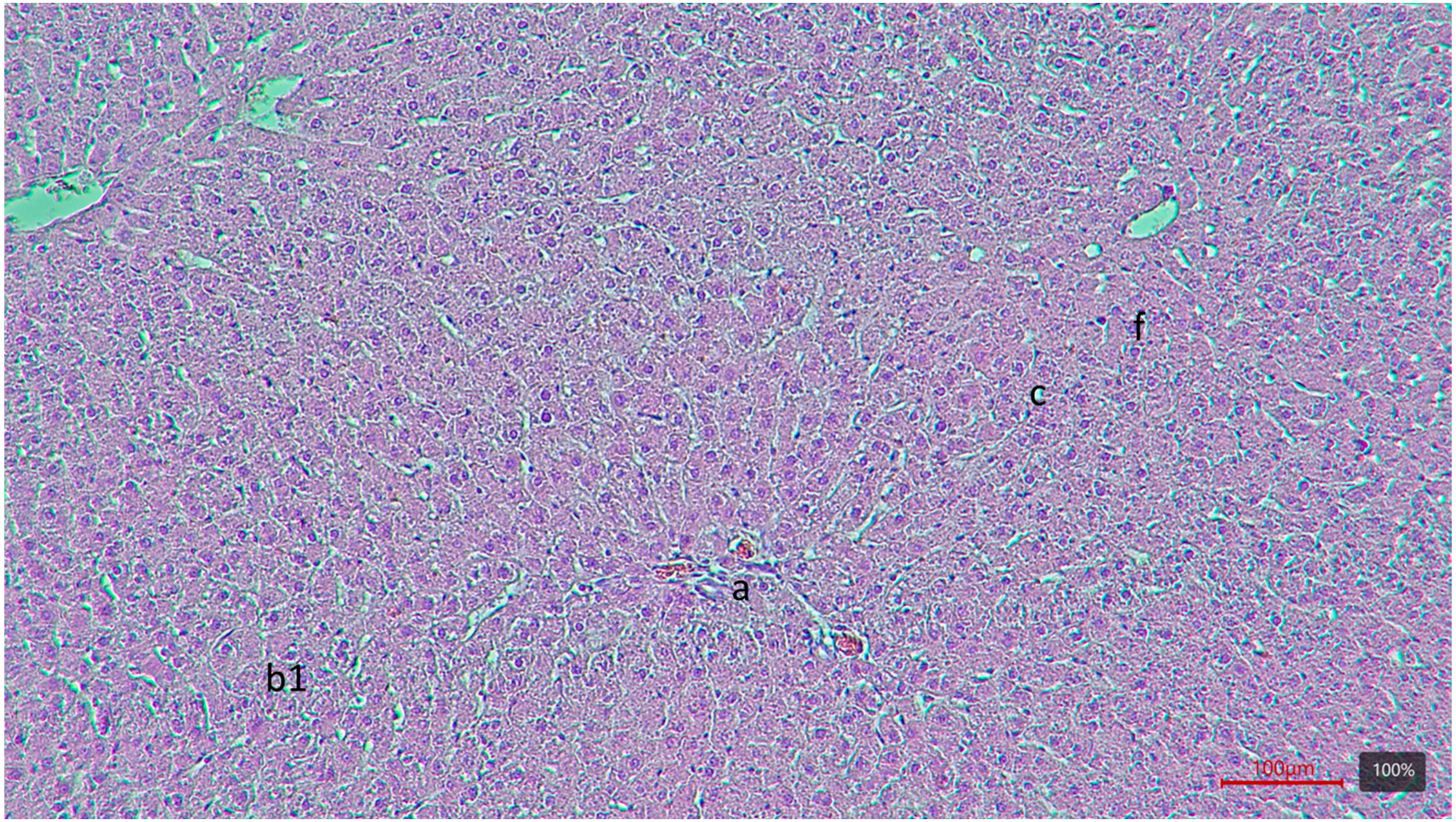

Figure S6. Micrograph of liver tissue sample **5**

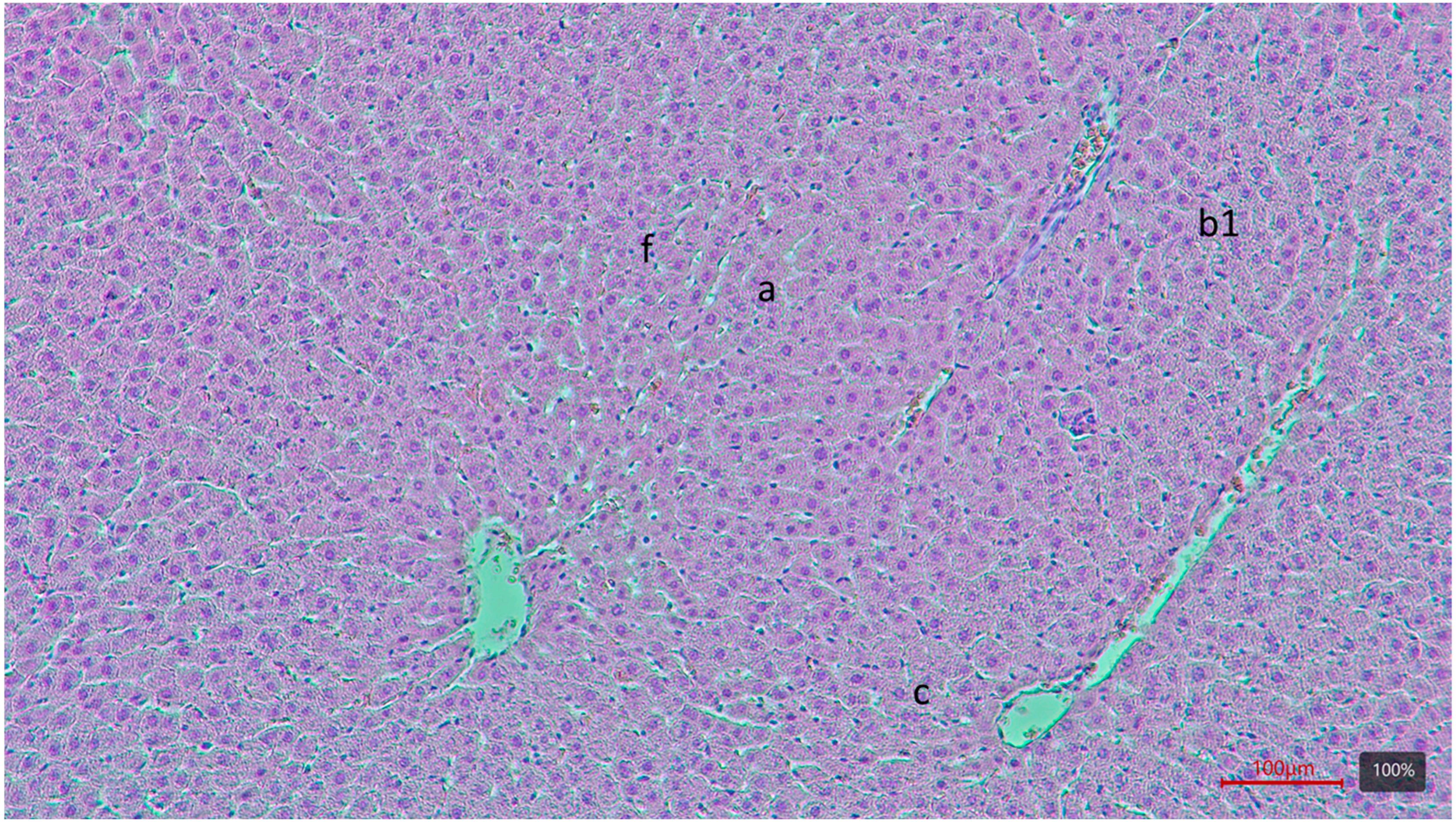

Figure S7. Micrograph of liver tissue sample **6**

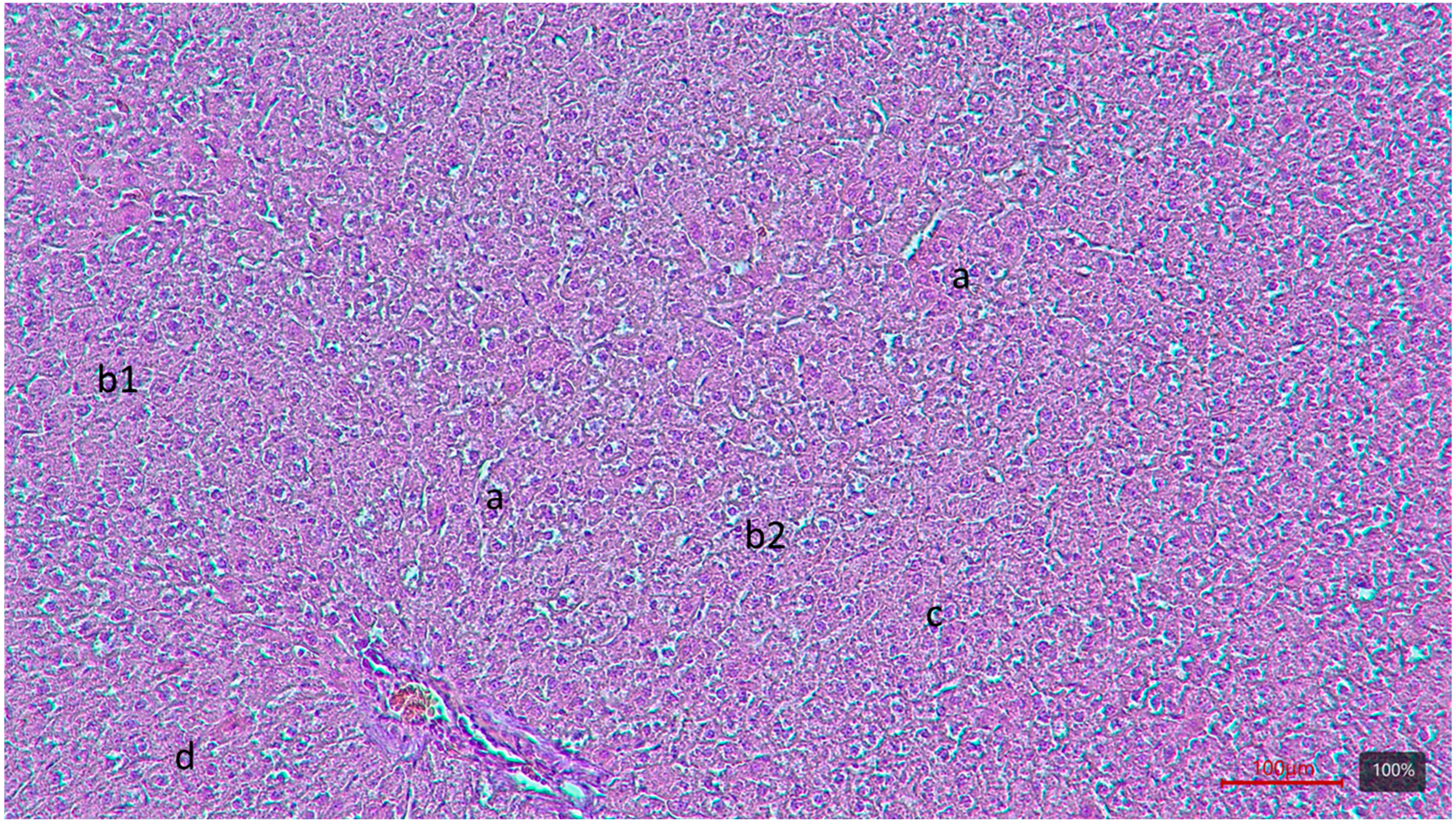

Figure S8. Micrograph of liver tissue sample **7**

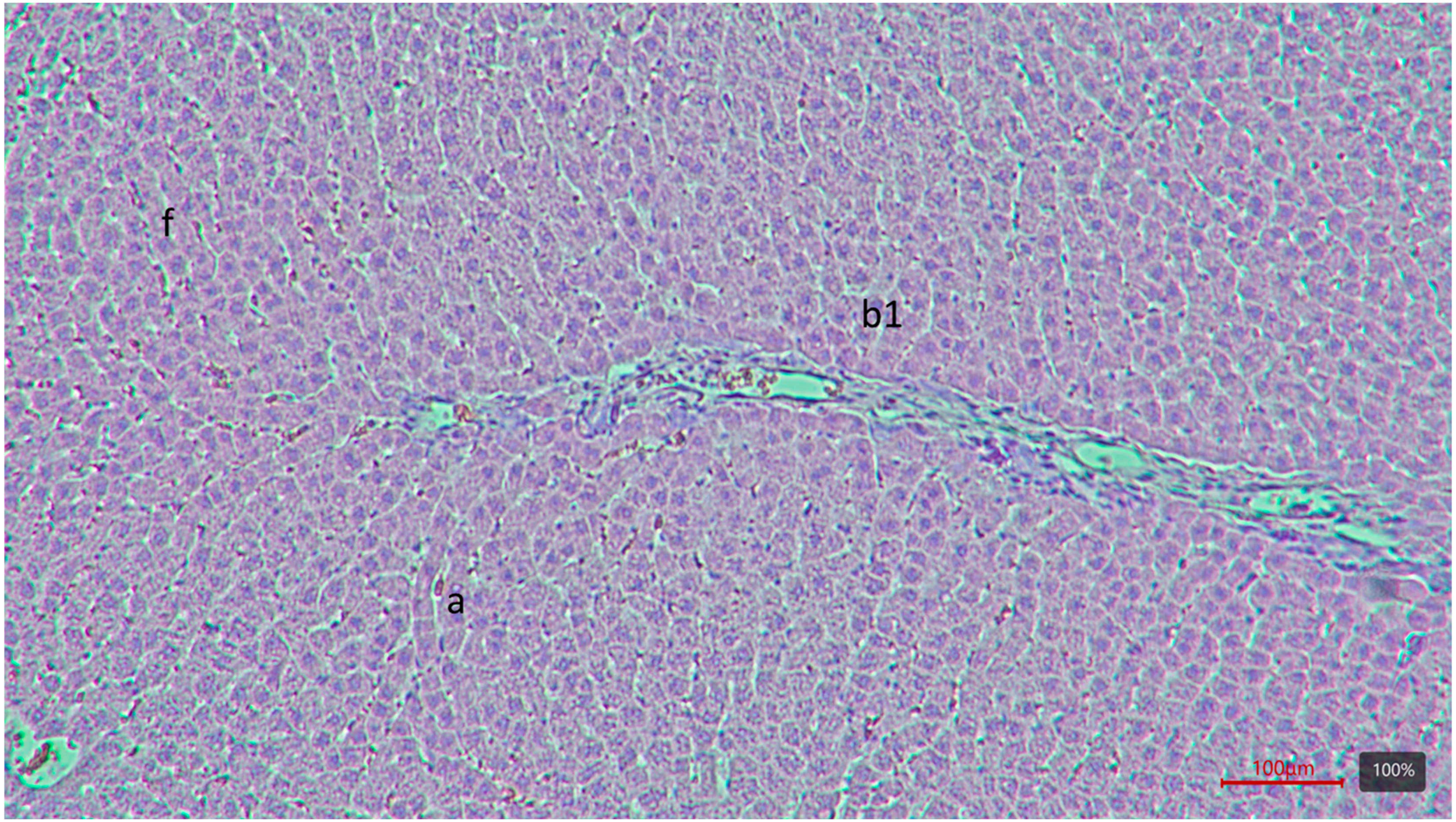

Figure S9. Micrograph of liver tissue sample **8**

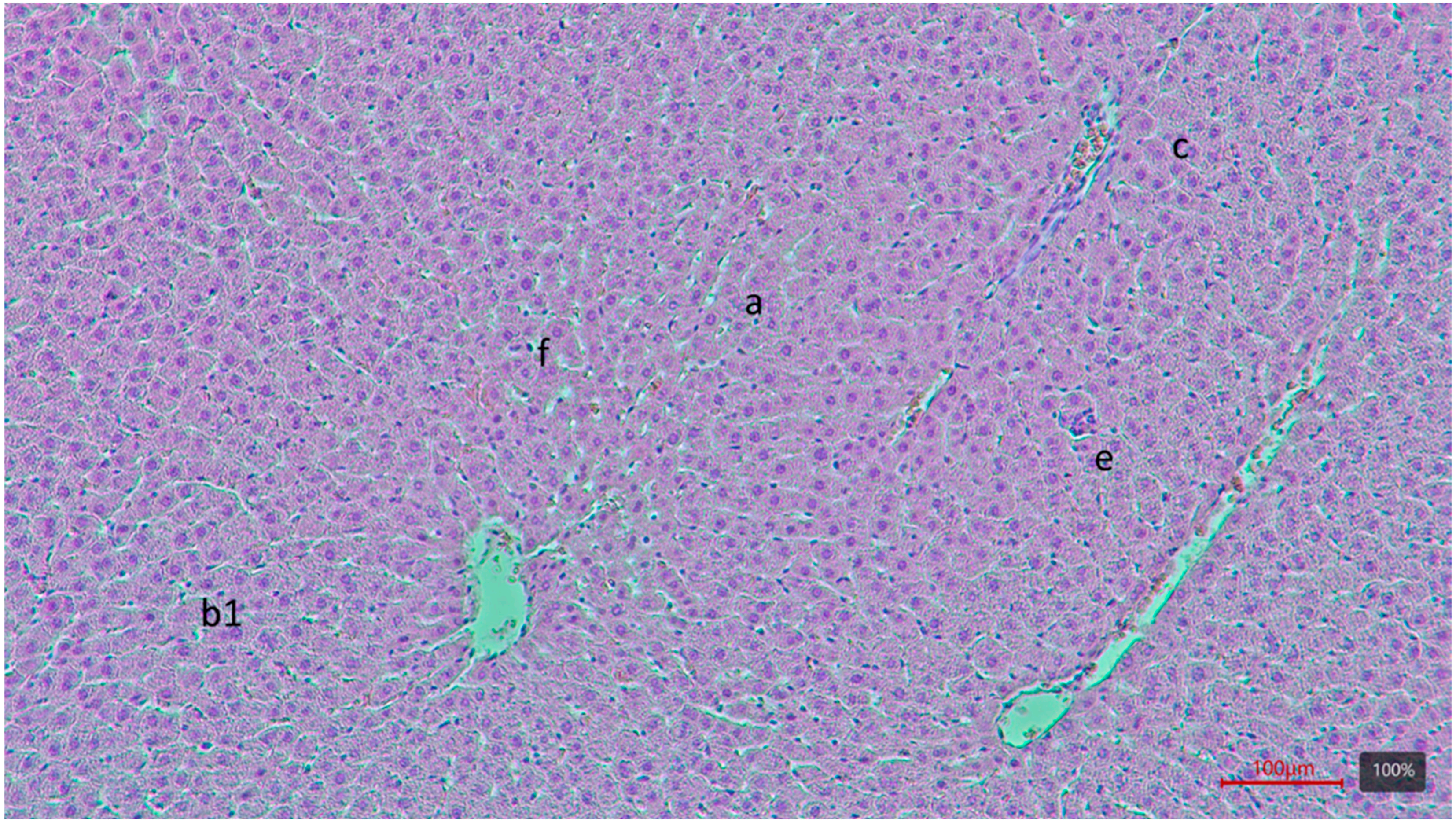

Figure S10. Micrograph of liver tissue sample **9**

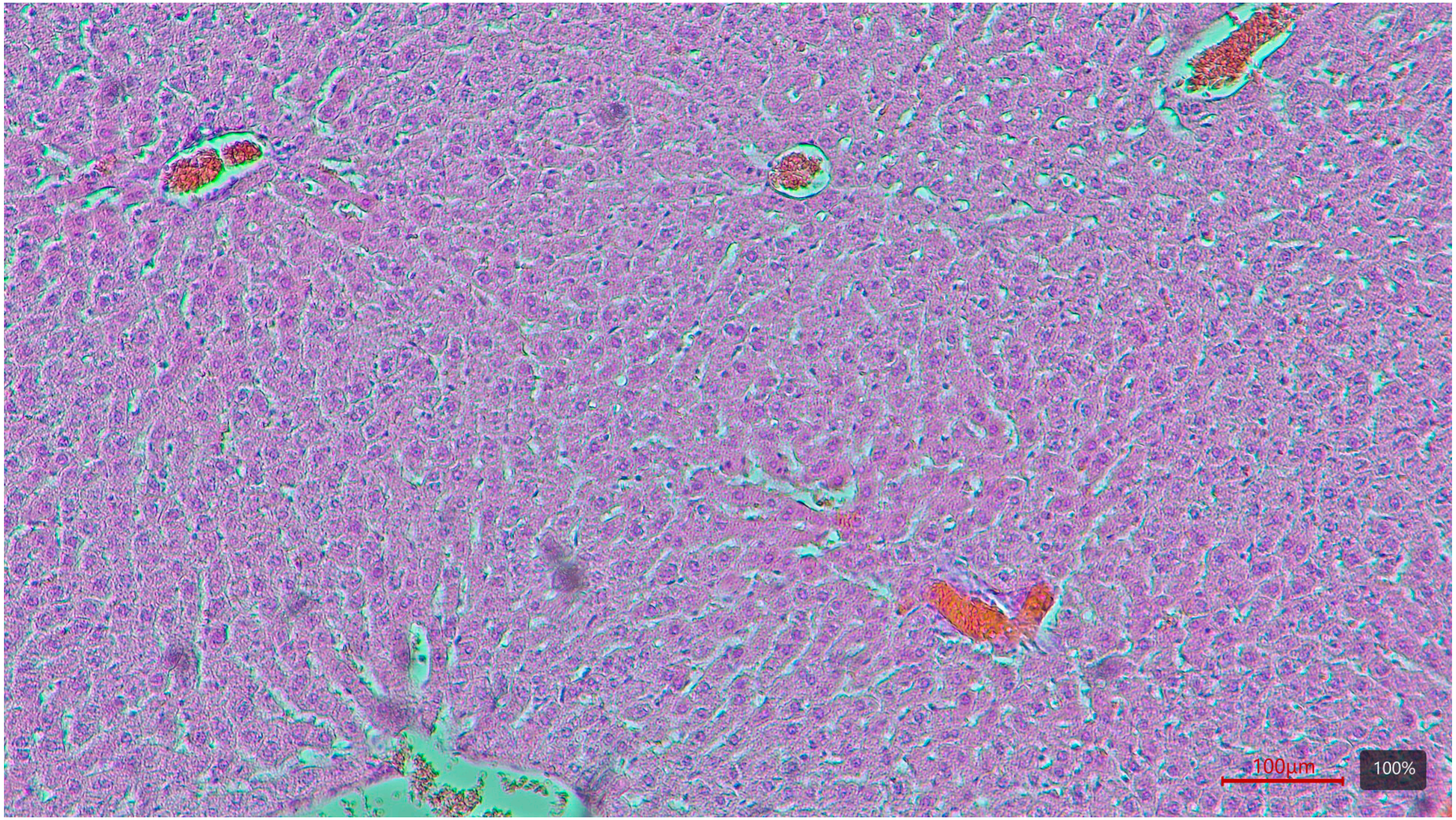

Figure S11. Micrograph of liver tissue of untreated rats (control)

1.2. Hystopatologycal analysis of kidney tissues

Labels indicate: a) Congestion, b) Glomerular atrophy, c) Hydrops degeneration, d) Necrosis of the cells, e) Atrophy and deskvamation, f) Eosinophilic material of tbi, g) Interstitial edema, h) Interstitial bleeding, i) Interstitial inflammatory infiltrate j) Changes in blood vessels

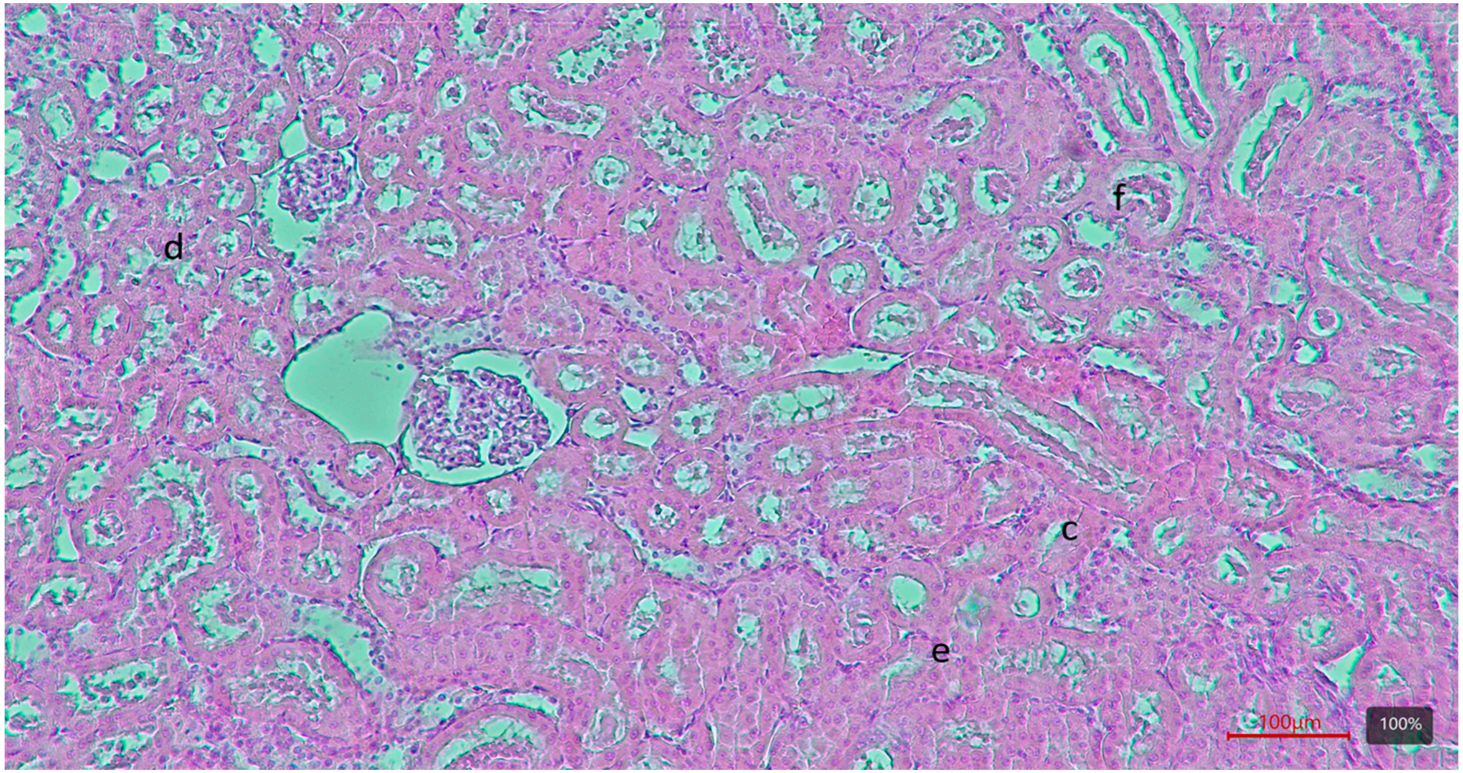

Figure S12. Micrograph of kidney tissues sample **0**

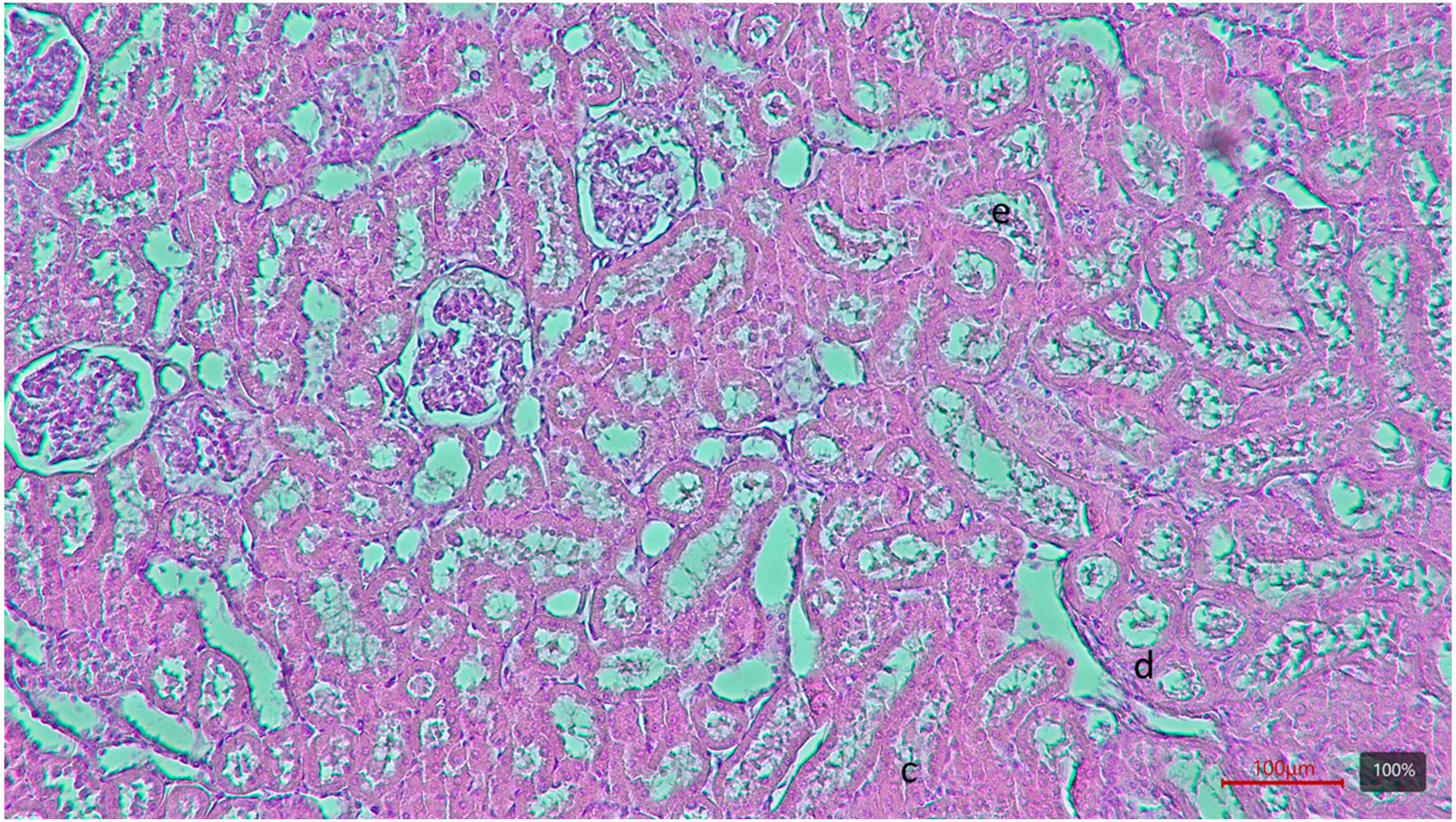

Figure S13. Micrograph of kidney tissues sample **1**

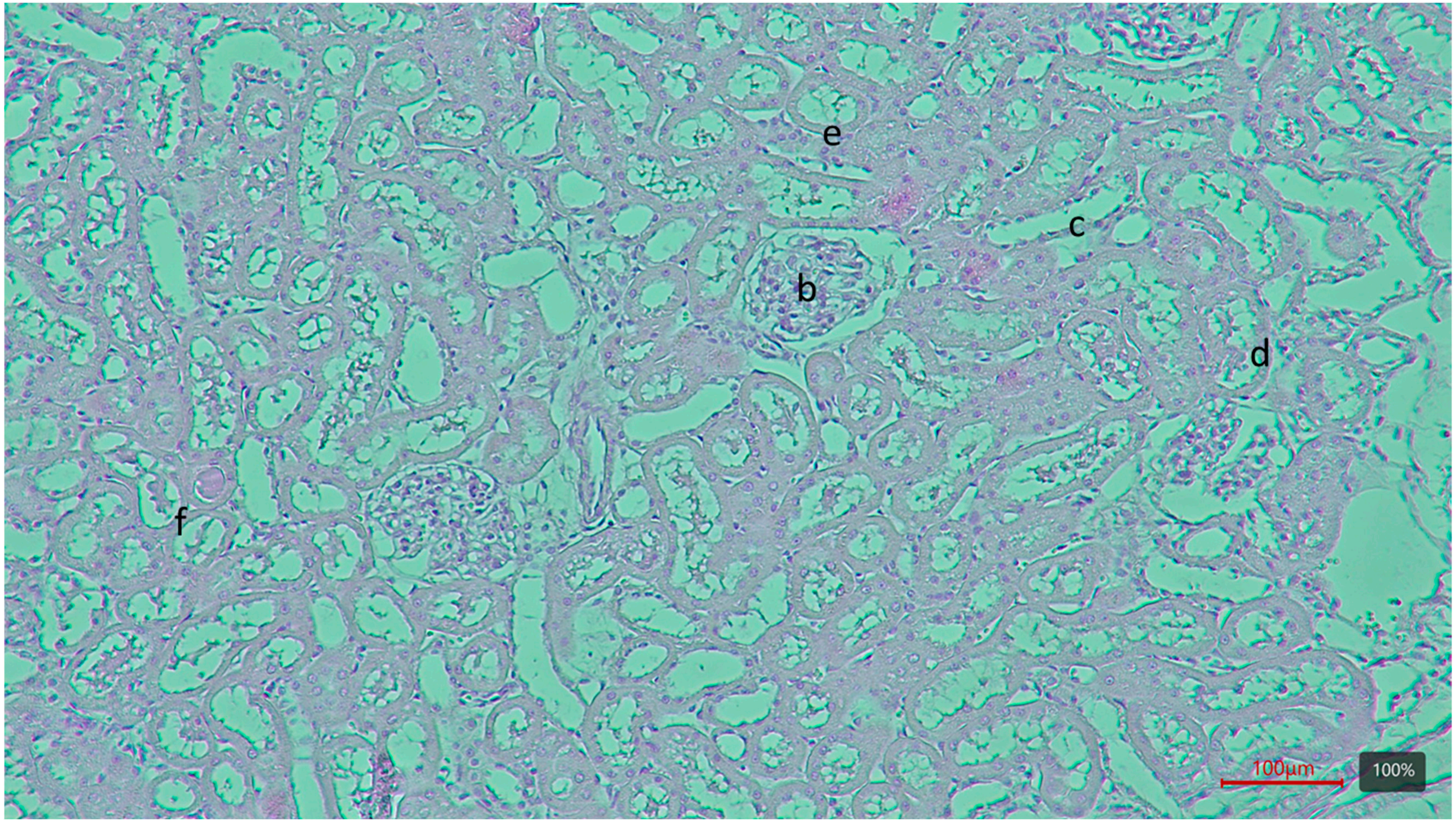

Figure S14. Micrograph of kidney tissues sample **2**

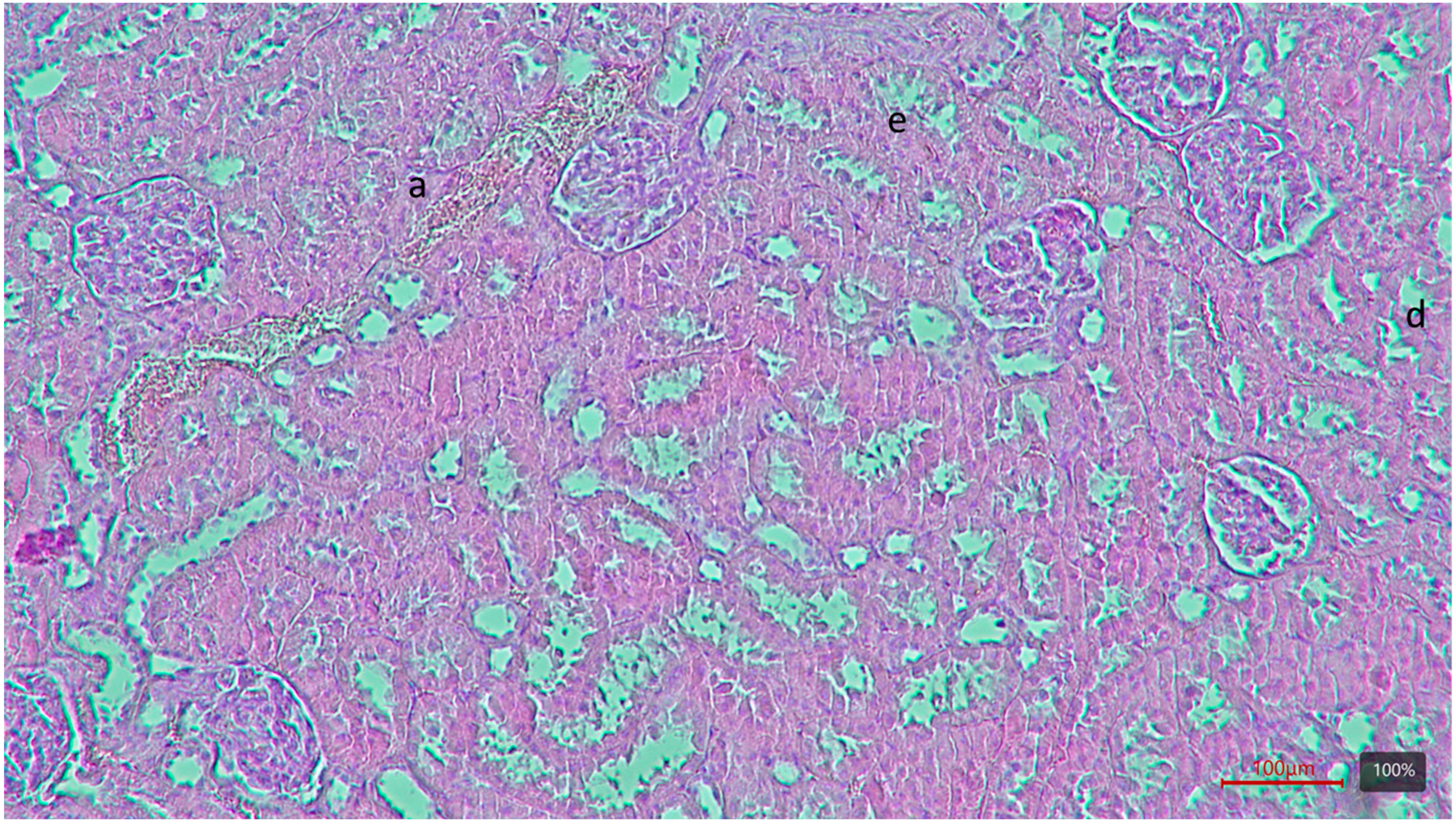

Figure S15. Micrograph of kidney tissues sample **3**

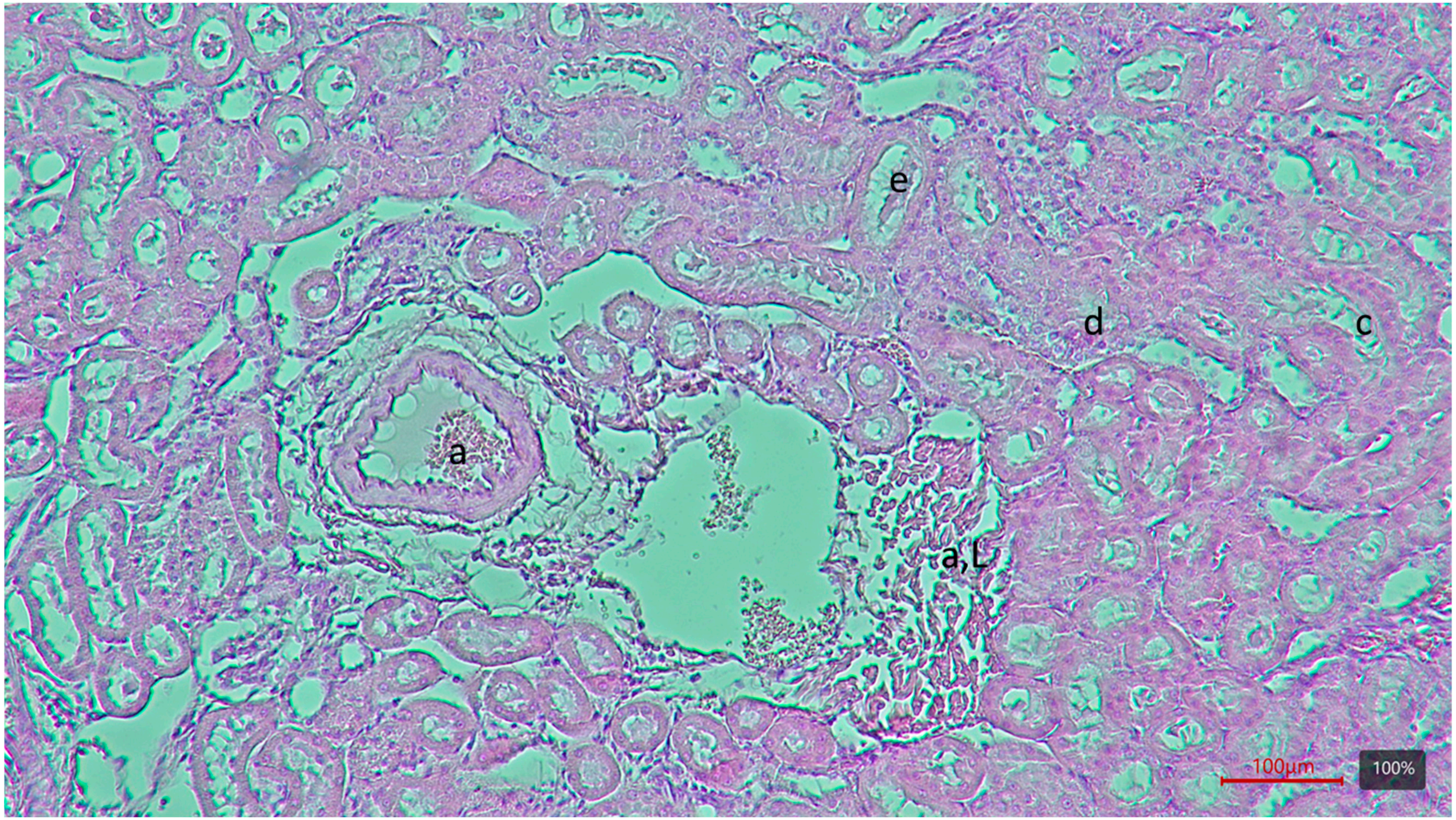

Figure S16. Micrograph of kidney tissues sample **4**

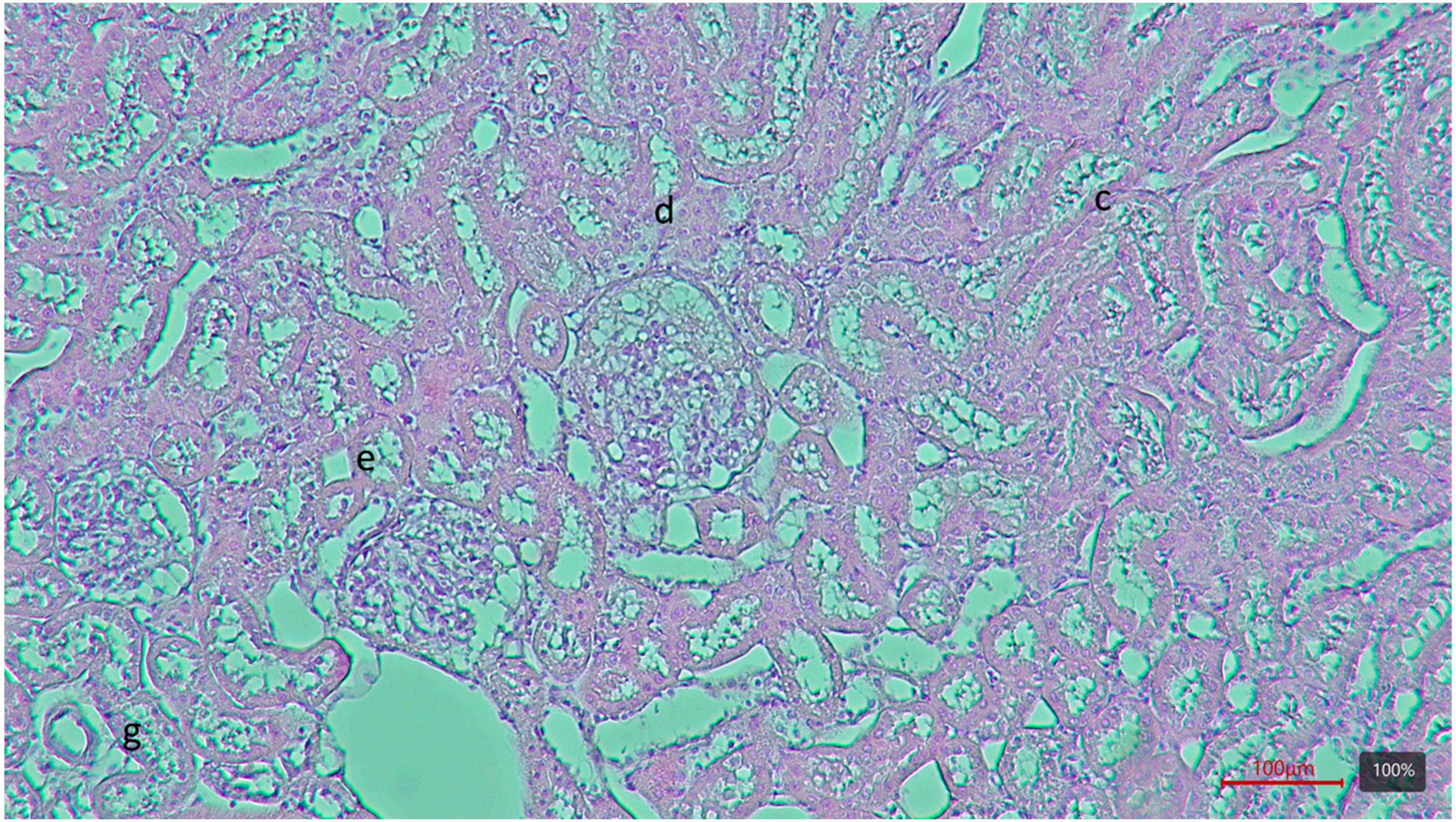

Figure S17. Micrograph of kidney tissues sample **5**

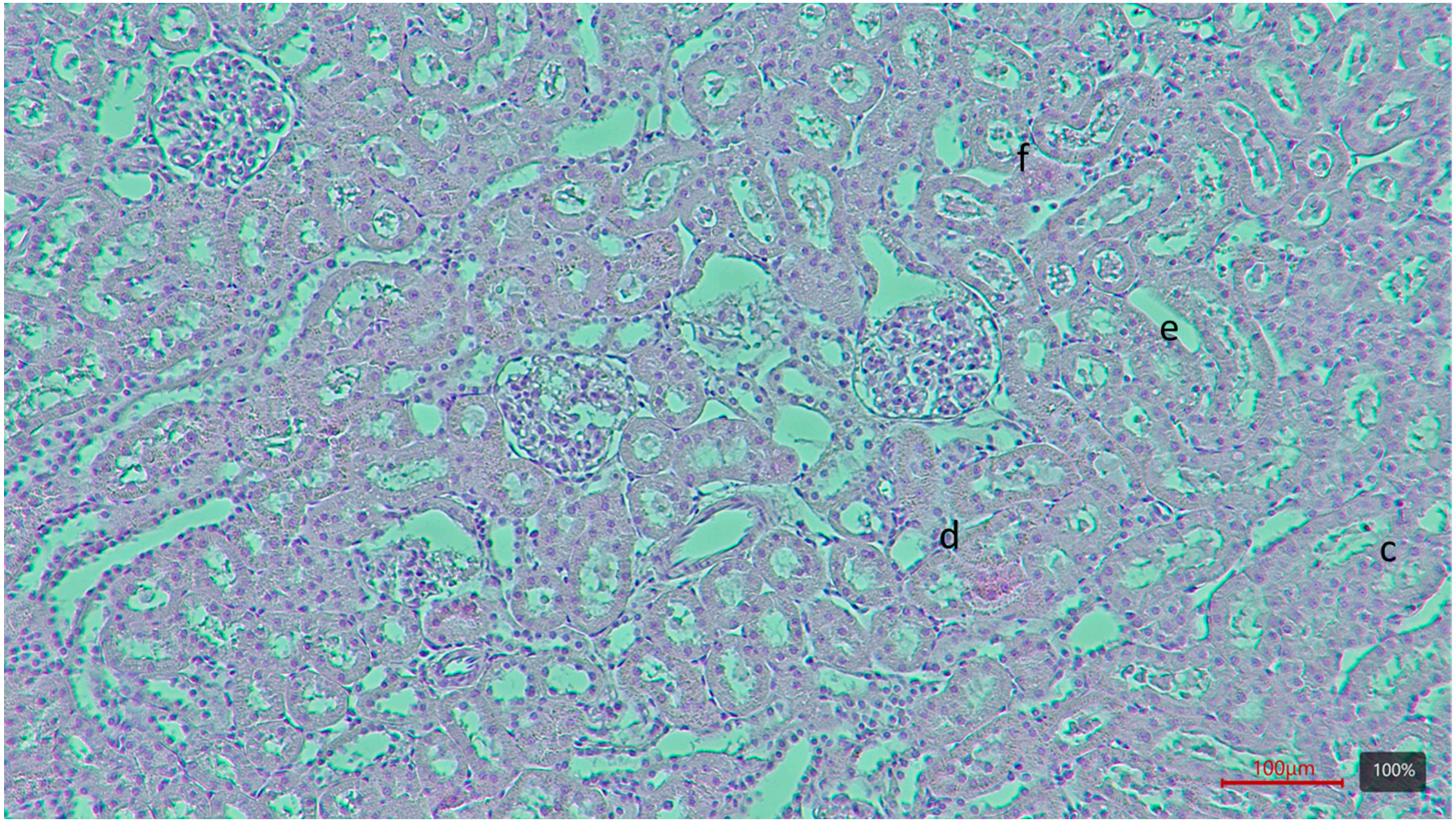

Figure S18. Micrograph of kidney tissues sample **6**

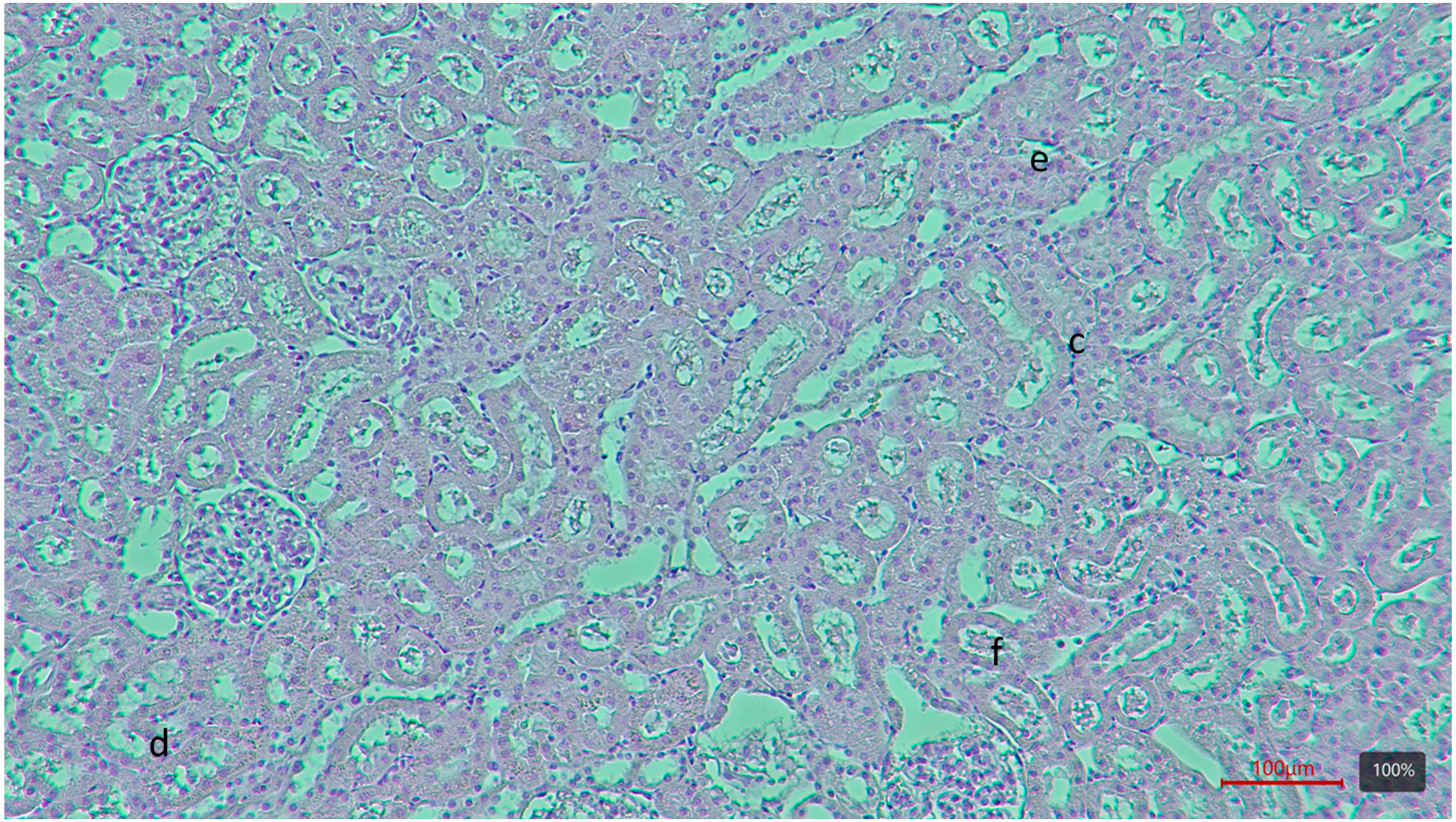

Figure S19. Micrograph of kidney tissues sample **7**

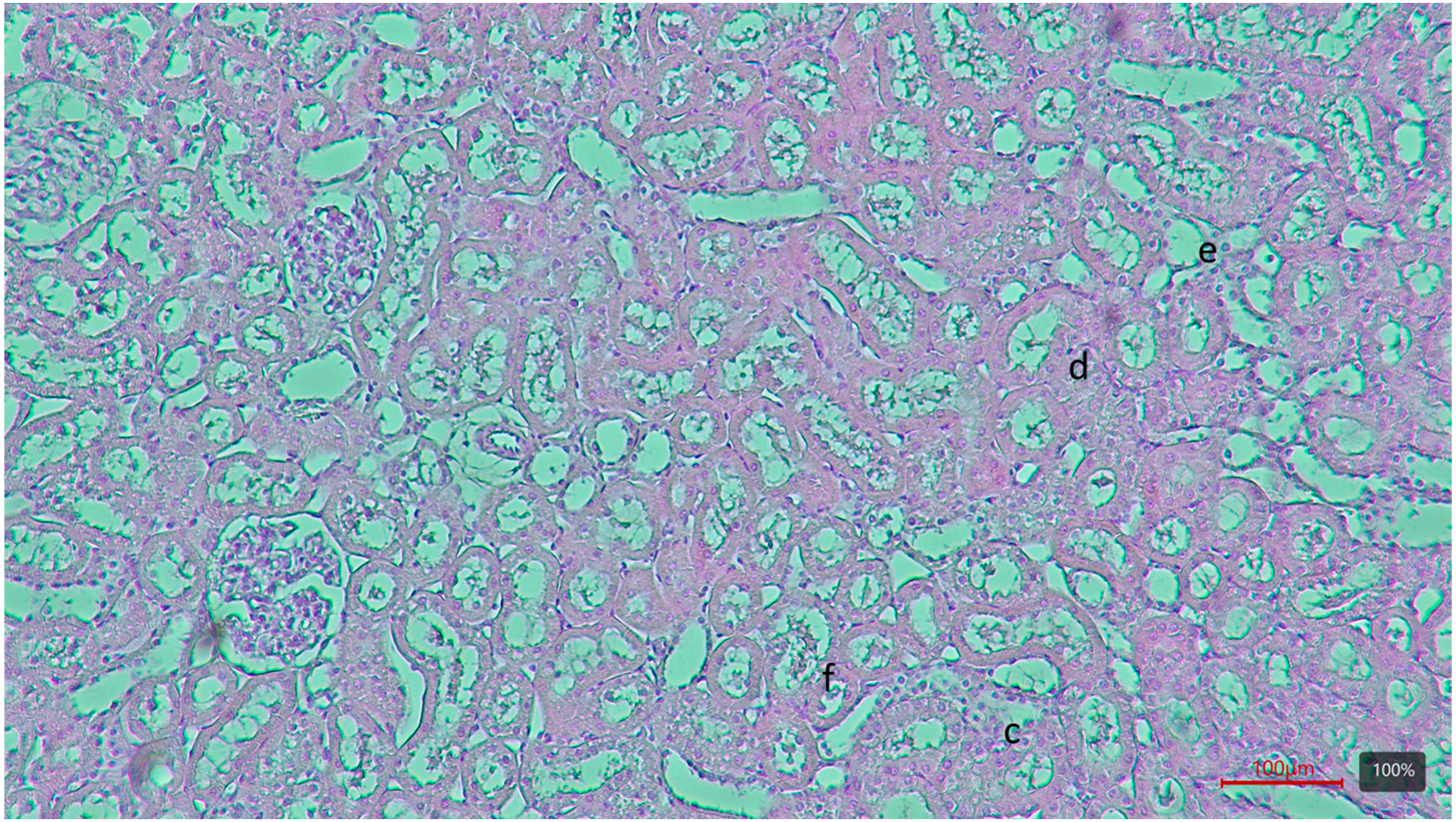

Figure S20. Micrograph of kidney tissues sample **8**

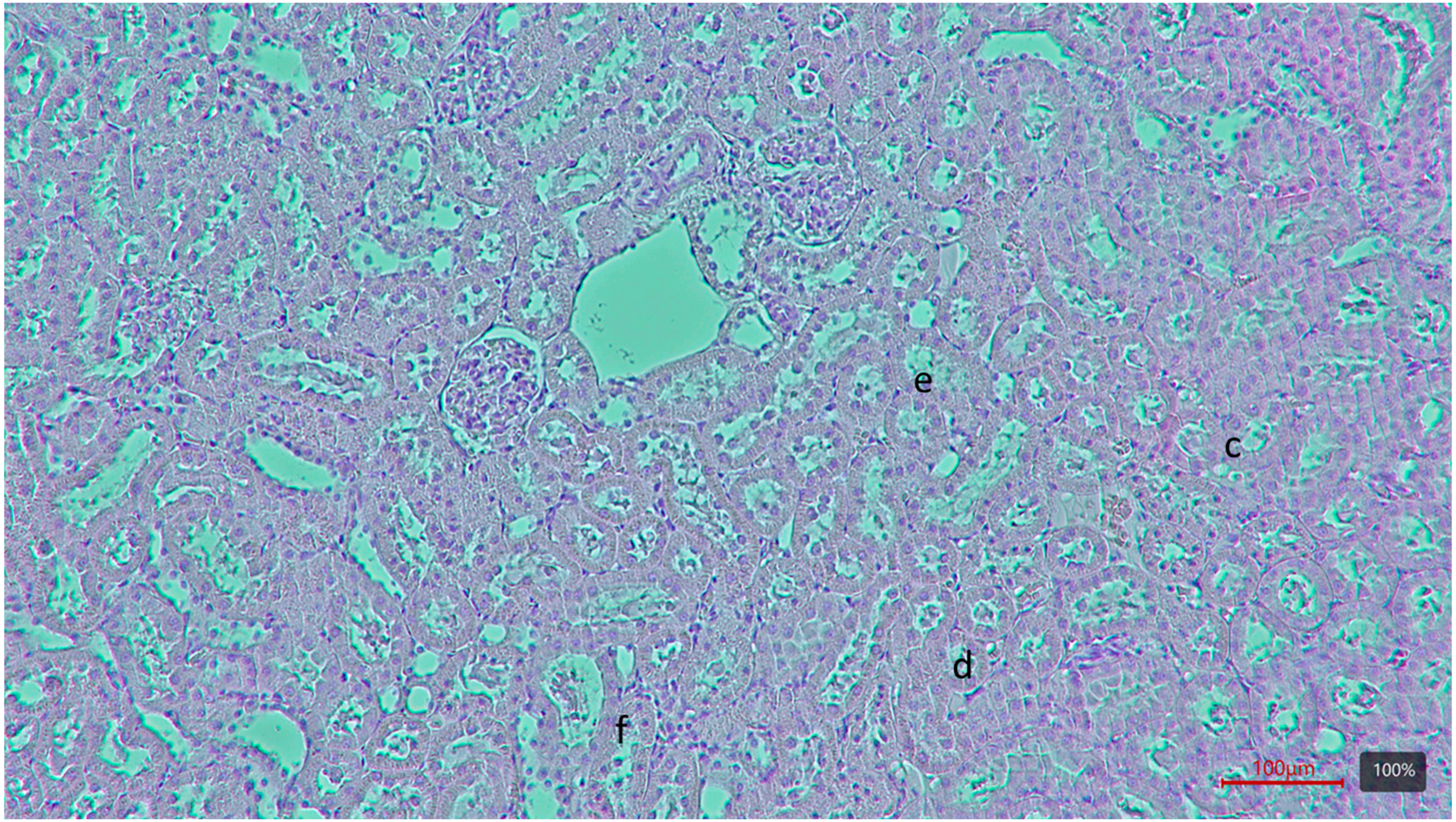

Figure S21. Micrograph of kidney tissues sample **9**

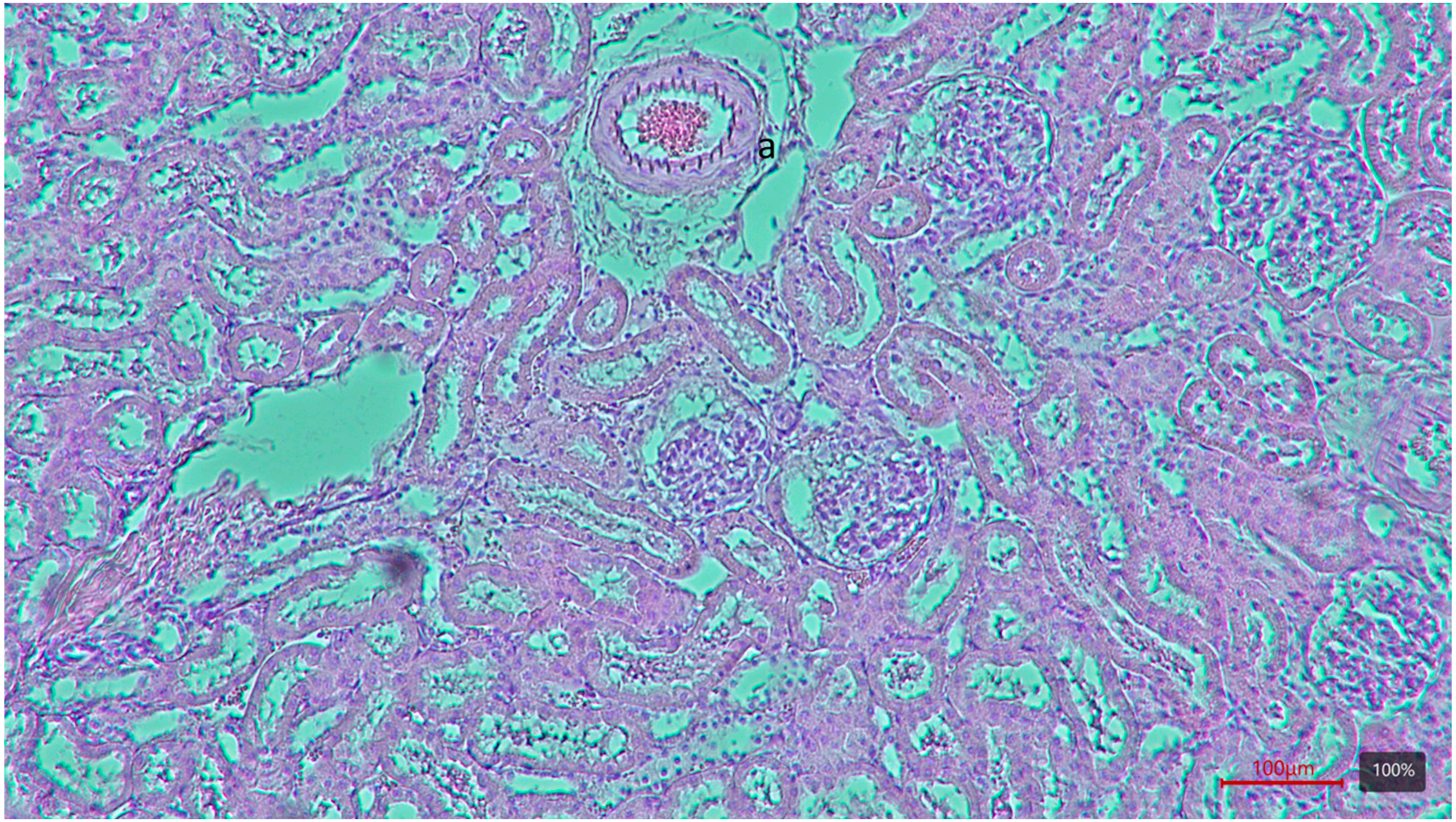

Figure S22. Micrograph of kidney tissues of untreated rats (control)

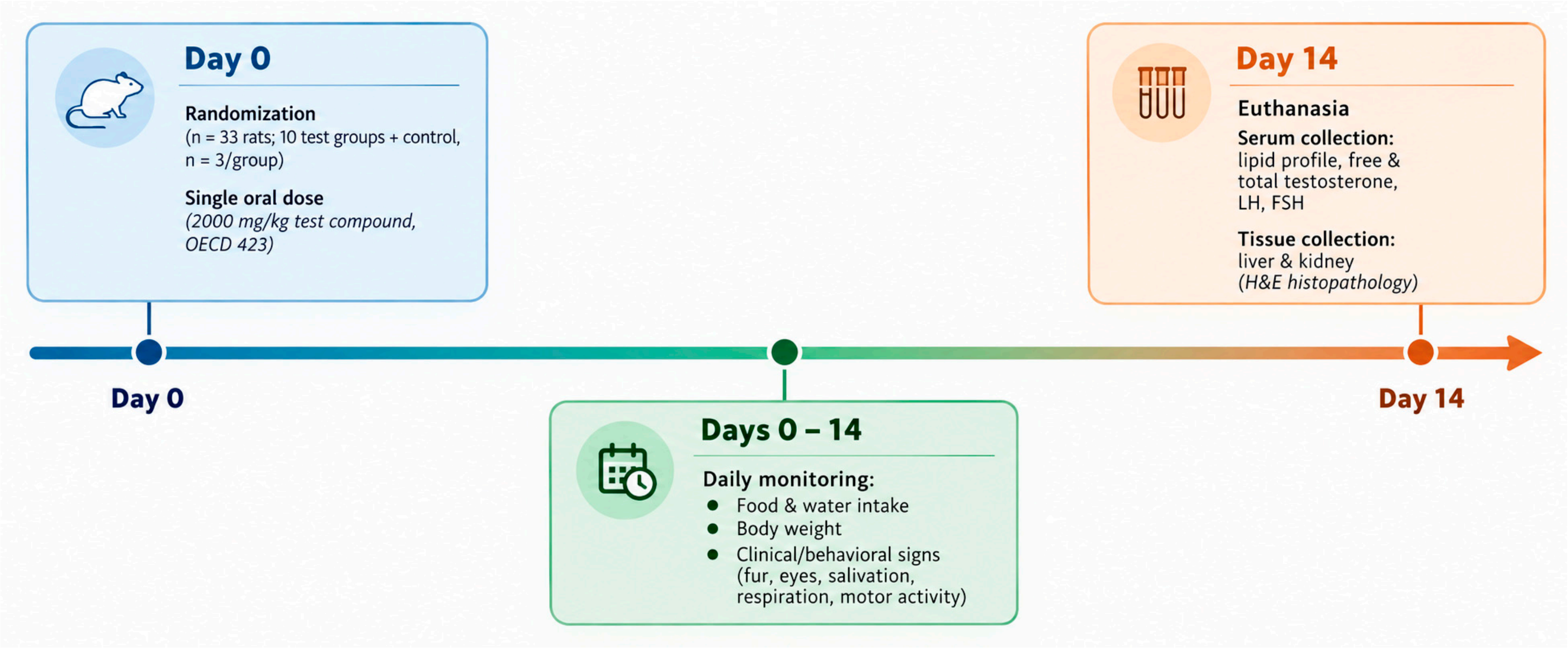

Figure S23. Experimental design of the 14-day acute oral toxicity study in rats

One particular batch of shilajit showed no measurable quantity and was chosen as reference blank. All validation experiments were done on this blank material spiked with analytical standards. Validation parameters were evaluated by analyzing all sample batches in triplicate for three consecutive days beginning with the 5-point calibration curve, reagent blank, analytical blank, check-standard (10 ppm) and fortified triplicates of three concentrations (1 ppm, 50 ppm and 100 ppm). The analytical blank for batch one and the second check-standard were injected last. Linearity was evaluated in the concentration range of 0.1-100 ppm. The coefficients of determination (R^2) ranged from 0.9960 to 0.9981. The limit of detection (LoD) was defined as the lowest concentration that could be integrated as a peak with a signal-to-noise ratio (S/N) of 3:1. LoQ was defined as the lowest concentration with a precision relative standard deviation (RSD) of <20% and signal-to-noise ratio (S/N) of not less than 10:1. Recovery was in the acceptable range of 84.3-102.5%.

Table S1. Calibration parameters

Standard	Y = a × X±b	R^2	LoD (µg kg^{-1})
**Uro-A**	Y=152980X+0	0.9996	52
**Uro-B**	Y=173282X+0	0.9992	35
**CA**	Y=8339.7X+0	0.9991	16
**FA**	Y=27267.2X+0	0.9991	27
**HCA**	Y=16049X+0	0.9996	16
**RA**	Y=15838X+0	0.9987	14

Table S2. Limit of quantification (LoQ) and recovery (%)

Element	LoQ (mg/kg)	Recovery (%)
As	0.004	102.0
Hg	0.001	101.3
Cd	0.001	99.8
Pb	0.004	96.3
Cu	0.066	98.9
Fe	0.23	99.1
Zn	0.372	98.5
Mn	0.011	101.9
Cr	0.007	103.8
Se	0.032	99.0
K	6.63	101.7
Na	16.76	99.0
Ca	9.24	99.2
Mg	0.4	101.5
Al	0.03	

Table S3. Semi-quantitative grading for liver and kidney tissues (all rats were evaluated); 0 = absent, 1 = minimal, 2 = mild, 3 = moderate, 4 = marked. The control column is the reference against which treatment-relatedness is judged.

**Lesion- Liver**	**Control**	**0**	**1**	**2**	**3**	**4**	**5**	**6**	**7**	**8**	**9**
Sinusoidal dilatation (a)	0	1	0	2	2	2	1	2	2	1	1
Hydropic degeneration (b1)	0	1	1	1	2	2	1	3	2	2	1
Ballooning degeneration (b2)	0	0	1	1	0	0	0	0	1	0	0
Focal necrosis (c)	0	1	1	1	1	2	2	2	1	0	1
Confluent necrosis (d)	0	0	0	1	0	0	0	0	1	0	0
Kupffer cell hyperplasia (e)	0	0	1	0	0	0	0	0	0	0	2
Lymphocyte infiltration (f)	0	0	0	0	1	0	2	1	0	1	2
Fibrosis (g)	0	0	0	0	0	0	0	0	0	0	0

**Lesion- Kidney**	**Control**	**0**	**1**	**2**	**3**	**4**	**5**	**6**	**7**	**8**	**9**
Congestion (a)	1	0	0	0	3	2	0	0	0	0	0
Glomerular atrophy (b)	0	0	0	2	0	0	0	0	0	0	0
Hydrops degeneration (c)	0	2	1	3	0	3	1	2	2	1	1
Necrosis of the cells (d)	0	2	1	3	3	3	2	1	2	1	3
Atrophy and deskvamation (e)	0	2	1	3	2	2	2	2	1	2	2
Eosinophilic material of tbi (f)	0	2	0	2	0	0	0	2	2	1	2
Interstitial edema (g)	0	0	0	0	0	0	2	0	0	0	0
Interstitial bleeding(h)	0	0	0	0	0	0	0	0	0	0	0
Interstitial inflammatory infiltrate (i)	0	0	0	0	0	0	0	0	0	0	0
Changes in blood vessels (j)	0	0	0	0	0	2	0	0	0	0	0
